# Ancient polymorphisms contribute to genome-wide variation by long-term balancing selection and divergent sorting in *Boechera stricta*

**DOI:** 10.1186/s13059-019-1729-9

**Published:** 2019-06-21

**Authors:** Baosheng Wang, Julius P. Mojica, Nadeesha Perera, Cheng-Ruei Lee, John T. Lovell, Aditi Sharma, Catherine Adam, Anna Lipzen, Kerrie Barry, Daniel S. Rokhsar, Jeremy Schmutz, Thomas Mitchell-Olds

**Affiliations:** 1 Key Laboratory of Plant Resources Conservation and Sustainable Utilization, South China Botanical Garden, Chinese Academy of Sciences, Guangzhou, 510650 China; 2 Department of Biology, Duke University, Box 90338, Durham, NC 27708 USA; 3 Institute of Ecology and Evolutionary Biology and Institute of Plant Biology, National Taiwan University, Taipei, 10617 Taiwan, ROC; 4 HudsonAlpha Institute for Biotechnology, Huntsville, AL 35806 USA; 5 Department of Energy Joint Genome Institute, Walnut Creek, CA 94598 USA

**Keywords:** Genomic diversity, Ancestral polymorphism, Balancing selection, Speciation

## Abstract

**Background:**

Genomic variation is widespread, and both neutral and selective processes can generate similar patterns in the genome. These processes are not mutually exclusive, so it is difficult to infer the evolutionary mechanisms that govern population and species divergence. *Boechera stricta* is a perennial relative of *Arabidopsis thaliana* native to largely undisturbed habitats with two geographic and ecologically divergent subspecies. Here, we delineate the evolutionary processes driving the genetic diversity and population differentiation in this species.

**Results:**

Using whole-genome re-sequencing data from 517 *B. stricta* accessions, we identify four genetic groups that diverged around 30–180 thousand years ago, with long-term small effective population sizes and recent population expansion after the Last Glacial Maximum. We find three genomic regions with elevated nucleotide diversity, totaling about 10% of the genome. These three regions of elevated nucleotide diversity show excess of intermediate-frequency alleles, higher absolute divergence (*d*_*XY*_), and lower relative divergence (*F*_*ST*_) than genomic background, and significant enrichment in immune-related genes, reflecting long-term balancing selection. Scattered across the genome, we also find regions with both high *F*_*ST*_ and *d*_*XY*_ among the groups, termed *F*_*ST*_*-*islands. Population genetic signatures indicate that *F*_*ST*_*-*islands with elevated divergence, which have experienced directional selection, are derived from divergent sorting of ancient polymorphisms.

**Conclusions:**

Our results suggest that long-term balancing selection on disease resistance genes may have maintained ancestral haplotypes across different geographical lineages, and unequal sorting of balanced polymorphisms may have generated genomic regions with elevated divergence. This study highlights the importance of ancestral balanced polymorphisms as crucial components of genome-wide variation.

**Electronic supplementary material:**

The online version of this article (10.1186/s13059-019-1729-9) contains supplementary material, which is available to authorized users.

## Background

How evolutionary processes drive genetic divergence and eventually lead to speciation is a fundamental question in evolutionary biology [1]. Taking advantage of next-generation sequencing technologies, heterogeneous genomic variation has been documented in many species [2–8], but disentangling factors shaping genomic landscapes remain challenging [9].

Balancing selection maintains multiple advantageous polymorphisms in populations and increases genetic diversity [10]. In contrast, positive and purifying selection favor single advantageous alleles and reduce genetic diversity [11, 12]. Balancing selection can persist for many generations, and maintains ancient polymorphisms in nascent species pairs, resulting in genomic regions with increased nucleotide diversity (*π*) in descendant species and low relative divergence (*F*_*ST*_) between species [13]. Alternatively, when selection varies geographically, it may favor locally adapted alleles in the nascent lineages [13]. In this case, ancestral balanced polymorphisms could be sorted unequally across descendant lineages by selection, generating genomic regions with both elevated *F*_*ST*_ and absolute divergence (*d*_*XY*_) [3, 7, 13]. Divergent sorting of ancient polymorphisms also could be facilitated by enhanced genetic drift as a consequence of population bottlenecks during speciation. Other processes can also generate highly differentiated regions (Table 1). During the process of isolation-with-migration, divergence might initiate in the regions with reduced gene flow and further extend to the surrounding areas due to the linked selection, resulting in genomic islands with elevated divergence (both *F*_*ST*_ and *d*_*XY*_) [3, 8, 11, 13]. Alternatively, forces such as background selection and recurrent selective sweeps tend to reduce genetic diversity in regions of low recombination, leading to elevated *F*_*ST*_ but unchanged or decreased *d*_*XY*_ [2, 4–6, 11]. These processes are difficult to discriminate because they are not mutually exclusive and could cause similar patterns in the genome (Table 1) [9]. Additionally, genetic drift and demographic processes may also be responsible for observed peaks of genomic diversity/divergence, mimicking the patterns produced by selection [14]. Therefore, inferring the evolutionary mechanisms that influence genomic landscapes requires detailed information on the speciation history and comparisons of lineages with contrasting divergence levels and geographic distribution [9].

**Table 1:** Predicted characteristics of genomic islands of divergence under different evolutionary models

Model	*F*_*ST*_	*d*_*XY*_	Polymorphism within populations	Local recombination rate	More islands in sympatry
Reproductive isolation, divergence with gene flow [3, 8, 13]	Elevated	Elevated	No prediction	No prediction	Yes
Local adaptation, divergence without gene flow [3]	Elevated	Not elevated	Reduced	No prediction	No
Recurrent selective sweeps within populations [2, 4–6, 11]	Elevated	Reduced	Reduced	Reduced	No
Background selection within populations [2, 4–6, 11]	Elevated	Reduced	Reduced	Reduced	No
Sorting of ancestral balanced polymorphisms [3, 7, 13]	Elevated	Elevated	No prediction	Unchanged or reduced	No

*Boechera stricta* (Brassicaceae), a perennial relative of *Arabidopsis thaliana*, is native to largely undisturbed sites in western North America [15–17]. Previous studies identified two subspecies (*East* and *West*) of *B. stricta*, with further subdivision within the *East* subspecies [18–21]. While genetic variation within subspecies is driven by geographic isolation [20], the divergence between *East* and *West* is significantly influenced by ecological adaptation [20]. These subspecies occupy different habitats, and *Western* genotypes are typically found in sites with more constant and abundant water supply, suggested that local water availability may be the selective force underlying ecological speciation between *East* and *West* subspecies [20]. In addition, greenhouse experiments revealed phenological and morphological traits under divergent selection between subspecies [19], and an *East*-*West* recombinant inbred line population segregates for many quantitative trait loci (QTLs) for ecologically important traits, including flowering time, herbivore resistance, fecundity, and lifetime fitness [21–24], which may have contributed to incipient ecological speciation in *B. stricta*. These characteristics, along with the sequenced genome [21], facilitate population genomic studies in *B. stricta* to understand how complex evolutionary forces drive divergence and speciation.

To understand how different evolutionary processes contribute to current genomic variation, we re-sequenced the whole genomes of 517 *B. stricta* accessions representing much of the species range. First, we investigated the population structure and history of species divergence. Next, we identified the signatures of long-term balancing selection influencing ~ 10% of the genome. Finally, we looked for genomic regions that distinguish lineages and assessed the roles of different evolutionary processes in driving divergence. Our study provides an example for disentangling the multitude of evolutionary processes that may have shaped the patterns of genetic variation across the genome and improves our understanding of the cause and consequence of genomic divergence during speciation.

## Results and discussion

**Fig. 1:**
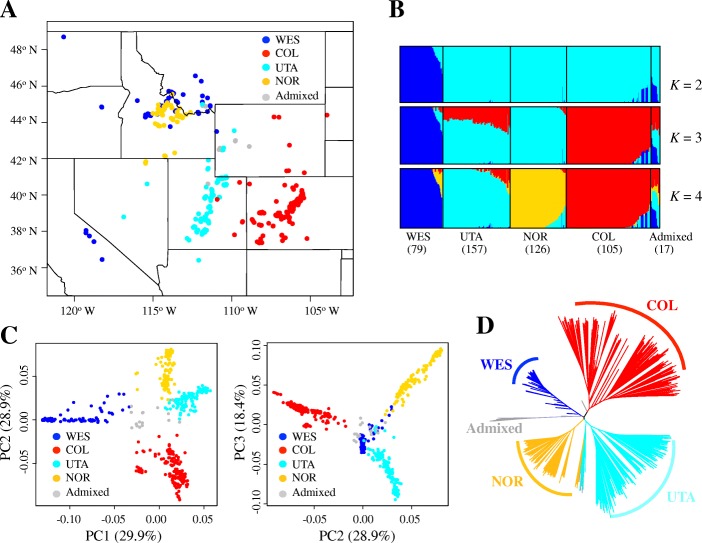
Geographic distribution and population structure. **a** Geographic distribution of the 484 *Boechera stricta* accessions. Each individual was assigned into one of the four genetic groups, WES (blue), COL (red), UTA (light blue), and NOR (gold), as well as Admixed (gray). **b** Population structure of *B. stricta* inferred by Admixture. Each vertical bar represents an individual, with different colors representing one of the genetic ancestries. The number of individuals in each lineage is also shown. *K* represents the number of structure groups for each analysis. **c** Genetic principal component analysis (PCA) of *B. stricta* based on genome-wide SNPs. Percent variation explained by each component is shown in parentheses. **d** Neighbor-joining (NJ) tree based on SNP data. Color scheme for genetic groups is the same in **a**–**d**

We performed whole-genome resequencing of 517 inbred *B. stricta* accessions using Illumina Hiseq2000/2500 short-read technology (Additional file 1: Table S1). Raw reads were mapped to the *B. stricta* reference genome v1.2 [21]. After quality control, 484 accessions with mean depth 5.05× were retained for subsequent analyses (Fig. 1a; Additional file 1: Table S1). We called SNPs using HaplotypeCaller in GATK v3.8 [25] and applied a series of stringent filtering criteria to identify a total of 4,125,395 high-quality SNPs (see “Methods” section for details). Comparison to Sanger sequences showed that the accuracy of our sequence calls exceeds 99.88% (Additional file 1: Table S2). To account for the uncertainty of genotypes called from short-read sequences, we estimated population genetic summary statistics based on genotype likelihoods as implemented in ANGSD [26].

### Discrete population structure and continuous genetic differentiation

We used Admixture [27], FastSTRUCTURE [28], sNMF [29], and NGSadmix [30] to infer individual ancestry. These four methods gave very similar results (Fig. 1b; Additional file 1: Figure S1) and detected clear population structure in *B. stricta*. The mode with *K* = 3 and 4 gave the highest Δ*K* values (Additional file 1: Figure S2); thus, we focus our analyses on the four genetic groups, WES, COL, UTA, and NOR (Fig. 1). With *K* = 4, we assigned each individual to one of the four groups if more than 50% of its genetic ancestry derived from the corresponding cluster (Fig. 1a; Additional file 1: Table S1). Seventeen individuals not matching this criterion were classified as “Admixed.” The four genetic groups showed a clear geographical distribution pattern (Fig. 1a). There is a little overlap among the groups, although NOR and WES have an area of sympatry in Montana and Idaho [21]. A principal component analysis (PCA) and a neighbor-joining (NJ) tree further confirmed the four genetic groups (Fig. 1c, d). High differentiation was detected among these four groups (Additional file 1: Table S3), similar to previous estimates based on microsatellite and low-copy nuclear DNA sequences [17, 18].

Genetic diversity in *B. stricta* consists not only of clusters, but also clines. Within groups, the geographic distribution of genetic variation is generally consistent with isolation by distance (IBD) models. We found significant correlations between pair-wise genetic distance and geographical distance within each group (*r* = 0.19–0.36, *P* = 0.0001–0.0023, 10,000 permutations in Mantel test; Additional file 1: Table S4), but the pattern of IBD varied among the groups (Additional file 1: Table S4), indicating different colonization or migration histories of these groups. In conclusion, we found both discrete population structure and continuous patterns of genetic differentiation in *B. stricta*; similar patterns have also been reported in humans [31] and *A. thaliana* [32].

### Population demography

#### Recent divergence and long-term small effective population size of *B. stricta*

**Fig. 2:**
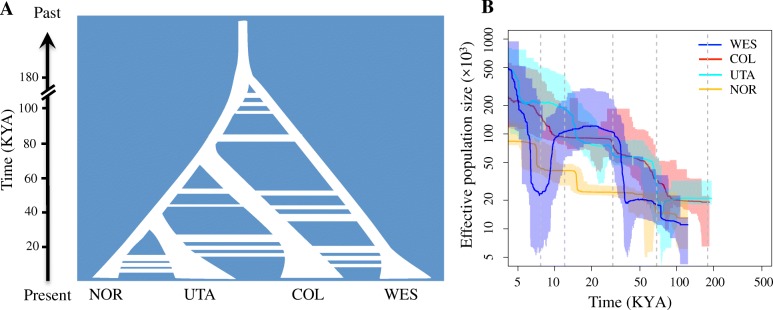
Demographic histories of *B. stricta*. **a** Schematic representing divergence processes inferred by fastsimcoal2. Estimated divergence time, population size, and gene flow are indicated here and detailed in Additional file 1: Figure S4 and Tables S6. **b** Changes of effective population size through time in the four groups. Solid lines represent means, and shading represents 95% percentiles of estimates. Vertical dashed lines mark times of population divergence and expansion estimated by fastsimcoal2

Estimates of demographic history and gene flow provide a sketch of population history and enable demographically informed simulations of population genetic variation under the assumption of selectively neutral evolution. Accordingly, we inferred the past demographic history of *B. stricta* from the joint site frequency spectrum (SFS) using coalescent simulation in fastsimcoal2 v.2.6.0.3 [33]. To avoid biases when determining the ancestral allelic states, we generated folded SFS following Excoffier et al. [33]. We used only fourfold degenerate sites and intergenic regions, as they are less affected by selection. Also, we removed the sites within the three genomic regions showing evidence of long-term balancing selection (see the “Balancing selection in *B. stricta* genomes” section). Fourteen demographic models were evaluated, considering a variety of scenarios for gene flow and population size changes (Additional file 1: Figure S3). The best-fit model (Model-11, Akaike’s weight of evidence ≈ 1, Additional file 1: Table S5) was a four-population isolation-with-migration model, where each group experienced two steps of population size changes after splitting (Fig. 2a; Additional file 1: Figure S4). By using a generation time of 2 years and mutation rate of 7 × 10^−9^ substitutions per site per year [34], we estimated the model parameters and their associated 95% confidence intervals (CIs) based on 100 parametric bootstraps (Additional file 1: Table S6). Notably, mutation rate and generation time are difficult to estimate and may vary over space and time. Consequently, inferred times and population sizes would need to be revised if these estimates were inaccurate.

In this best-fit model, the estimated divergence time between the two subspecies (WES group and ancestor of three *East* subspecies groups) was ~ 179 thousand years ago (KYA; 95% CI = 154–185 KYA), and divergence within the *East* subspecies dates to 30–70 KYA (Fig. 2a; Additional file 1: Figure S4; Additional file 1: Table S6). Historical effective population size (*N*_e_) was small in *B. stricta*; the estimated *N*_e_ values for the common ancestor of all groups, ancestor of *East* subspecies, and ancestor of UTA and NOR are 2.4 × 10^4^, 3.6 × 10^4^, and 3.9 × 10^4^, respectively. After splitting, the WES and NOR groups both had small initial populations (*N*_e_ = 1.9 × 10^4^ and 1.1 × 10^4^, respectively) comparable with their ancestors, while the COL and UTA groups had relatively larger initial populations (*N*_e_ = 1.0 × 10^5^ and 1.1 × 10^5^, respectively). The long-term small effective population sizes of WES and NOR are consistent with the relatively high level of linkage disequilibrium (LD) (Additional file 1: Figure S5) and low level of nucleotide diversity in these two groups (Additional file 1: Table S7). All groups experienced rapid population expansion at ~ 12 KYA and more recent exponential growth starting at ~ 7.7 KYA (CI = 5.0–9.7 KYA). Inferred gene flow was low among the groups (per generation migration rate = 6.8 × 10^−10^–2.0 × 10^−5^; Additional file 1: Table S6), which is expected given frequent inbreeding [17], low seed dispersal, and substantial geographic distances in *B. stricta* [15].

#### Validation of demographic inference

Evaluating null hypotheses of neutral evolution requires realistic population models, so we used two approaches to validate our demographic inferences. First, we evaluated the goodness-of-fit of the best model by comparing SFS and two summary statistics (*π* and *F*_*ST*_) between observed and simulated data. We found that SFS and summary statistics predicted under neutrality are well-matched to the data (Additional file 1: Figure S6). Second, to avoid limitations of model-based demographic inference, we also employed a model-flexible Stairway plot v.2 [35] method to investigate the recent fluctuation of *N*_e_, based on the folded SFS. In general, these results are consistent with those from fastsimcoal2. All groups showed lower ancestral population size (1 × 10^4^–2 × 10^4^) and recent population expansions within 15 KYA (Fig. 2b). In summary, validation analyses suggested that the best-fit model captures major aspects of the demographic history of our populations from patterns of genetic diversity. We applied this model in testing the significance of the outliers in subsequent analyses.

### Balancing selection in *B. stricta* genomes

#### Long-term balancing selection affected 10% of the *B. stricta* genome

**Fig. 3:**
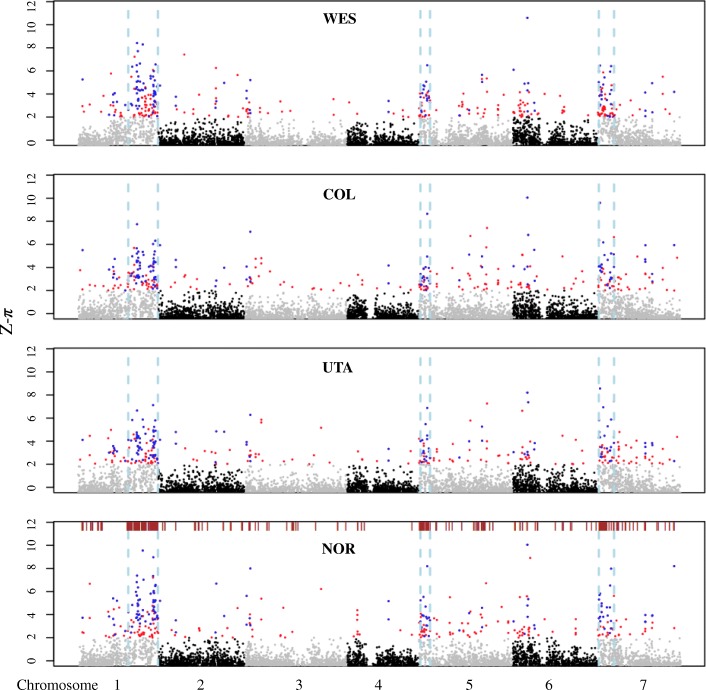
Genome-wide pattern of nucleotide diversity. *Z*-transformed nucleotide diversity (*Z*-*π*) is plotted along the genome with 20-kb non-overlapping windows for each group. The 130 outlier windows shared by all groups are shown in blue, and other islands specific to group(s) are in red. The dashed vertical lines (light blue) delimit three highly polymorphic genomic regions enriched for NBS-LRR genes. NBS-LRR genes are indicated by brown-colored bars above the Manhattan plot of NOR group. Alternating colors paint the different chromosomes, and windows with *Z*-*π* < 0 are not shown

In order to reveal evolutionary processes driving genomic variation, we estimated nucleotide diversity (*π*) in 20-kb non-overlapping windows across the genome. Then, we computed *Z*-transformations (*Z*-*π*) separately in each group (see the “Methods” section for details). This transformation puts the four genetic groups on the same centered, relative scale of nucleotide diversity. Genomic regions with *Z*-*π* ≥ 2 (corresponding to 4.30% of windows from all comparisons) were identified as outlier windows (Fig. 3). Within the four genetic groups, we detected 307–345 outlier windows for *Z*-*π* (“*π*-islands”; Additional file 1: Table S7).

Among these outlier windows, 130 were shared by all groups (henceforth referred to as shared outliers; Fig. 3), which could be generated by shared evolutionary forces influencing particular genomic regions. These *π*-islands were unevenly distributed in the genome and clustered into three genomic regions. These three regions were henceforth referred to as “Balancing selection (BLS) regions” (Fig. 3; see the “Methods” section for delimiting the BLS regions). BLS regions, with a total length of ~ 20 Mb (~ 10% of the 196.5 Mb assembled genome) [21], contain about half of the *π*-islands (46–49%) including 91 (70%) shared outliers (Additional file 1: Table S8).

All three BLS regions showed nucleotide diversity 2.57–3.88 times higher than the genomic background (*P* < 10^−5^, randomization test; Additional file 1: Table S9). Comparison of demographically informed neutral simulations with observed molecular variation suggests that non-neutral evolutionary processes have contributed to the elevated diversity in these regions (Additional file 1: Figure S7). The high diversity in BLS regions could be maintained by long-term balancing selection [10], but could also be due to the systematic variation in mutation rate, recombination rate, or gene density [12]. We checked each of these possible influences. First, to correct for variation in mutation rate among genomic regions, we divided diversity by divergence to an outgroup species, *Boechera retrofracta* [21, 36]. These outgroup-adjusted diversity levels are still higher in the BLS regions compared to genomic background (*P* < 10^−5^, randomization test; Additional file 1: Table S9). Therefore, the high diversity in genomic islands is not attributable to the elevated mutation rates. Second, to assess the impact of recombination rate on genetic diversity, we estimated the population-scaled recombination rate (*ρ* = 4*N*_e_c) in 20-kb windows across the genome using LDhelmet [37]. Following Wang et al. [4], we divided *ρ* by genetic diversity (*π*) to account for the confounding effects of local *N*_e_ and compared scaled *ρ* (*ρ*/*π*) between the BLS regions and the rest of the genome. Relative to the genomic background, we found similar recombination rate in the BLS regions of groups WES and NOR (scaled *ρ*: 0.25–0.29 vs. 0.24, *F*_3,7221_ = 1.03, *P* = 0.377 in WES; 0.16–0.26 vs. 0.36, *F*_3,6676_ = 2.42, *P* = 0.064 in NOR) but significant higher and lower recombination in the BLS regions of groups COL and UTA, respectively (scaled *ρ*: 0.73–0.90 vs. 1.15, *F*_3,7198_ = 5.76, *P* = 0.0006 in UTA; 0.77–1.13 vs. 0.59, *F*_3,7287_ = 34.77, *P* < 0.0001 in COL; Additional file 1: Table S10). It is possible that other historical factors, such as population structure, might explain the patterns in COL and UTA. For example, we estimated the recombination rate in each subgroup of COL and found similar estimates of scaled *ρ* between the BLS regions and background in one subgroup with low differentiation (0.64–0.81 vs. 0.65, *F*_3,7233_ = 2.25, *P* = 0.080; Additional file 1: Table S10). To further evaluate the effects of recombination rate on genetic diversity, we simulated a variety of recombination rates from 0 to 250 cM/Mb. We found that estimated diversities in all simulated data were significantly lower than the observations in the BLS regions (*W* ranges from 22,399,000 to 45,812,000, *P* < 2e^−16^, Mann-Whitney *U* test; Additional file 1: Figure S7). These results suggest that the recombination rate might have a little effect on the patterns of nucleotide diversity in *B. stricta*. A previous study in *A. thaliana* also revealed that regions collinear to these BLS regions showed recombination rates close to the genome-wide average [38]. Third, we compared gene density (mean length of coding sequences per 20-kb window) in outlier windows versus genome-wide. Gene density in two (LG1p and LG7p) of the BLS regions is slightly lower than the genomic background (3.93 kb vs. 4.56 kb, *P* < 0.001 for LG1p; 3.73 kb vs. 4.56 kb, *P* = 0.0016 for LG7p; randomization test; Additional file 1: Table S9) but is not different from the genomic background in LG5p (*P* = 0.253, randomization test; Additional file 1: Table S9). Additionally, controlling for recombination rate and gene density, the scaled diversity in the BLS regions is still higher than the genomic background (*F*_1,6217–6998_ = 307.12–1525.90, *P* < 2e^−16^; Additional file 1: Table S11). These results suggest that a high recombination rate or low gene density is unlikely to generate genomic islands of high diversity. Therefore, our results suggest that these regions reflect long-term balancing selection [10]. The hypothesis of long-term balancing selection was also supported by an excess of intermediate-frequency alleles (higher Tajima’s *D*, and Fay and Wu’s *H*) and lower population differentiation (*F*_*ST*_) in the BLS regions compared to the genomic background (Additional file 1: Table S9). In comparison with genome-wide averages, these regions showed higher absolute divergence (*d*_*XY*_) and also relative node depth (RND [39]; Additional file 1: Table S9), which takes into account varied mutation rate across the genome by dividing *d*_*XY*_ of each group pair with their mean divergence to an outgroup species, *B. retrofracta.* These results may reflect higher levels of ancestral polymorphism prior to the split of these groups [11].

#### Long-term balancing selection in disease resistance genes

Plant disease resistance genes, such as nucleotide-binding site-leucine-rich repeat (NBS-LRR) gene family, can be targets of balancing selection, and genetic variation in these gene regions could be maintained for long periods by transient or frequency-dependent selection [40]. While NBS-LRR genes have not been functionally characterized in *B. stricta*, previous studies have found several NBS-LRR genes under balancing selection in closely related species, e.g., *A. thaliana* [41, 42] and *Capsella* [43]. In the *B. stricta* genome [21], we identified 378 genes that were homologous to members of NBS-LRR gene family of *A. thaliana* [44], with 277 (60%) of them located in the BLS regions—a sixfold enrichment of NBS-LRR in BLS regions (Additional file 1: Table S8). Strong positive correlations (Spearman’s *ρ* = 0.19–0.41, *P* < 0.001; Additional file 1: Figure S8) between density of NBS-LRR genes and scaled diversity suggest long-term balancing selection on NBS-LRR genes and may have maintained genetic diversity in BLS regions. To avoid ambiguous alignments due to rapid evolution of NBS-LRR genes [44], we excluded NBS-LRR gene regions (4.8% of the three genomic regions) from the data analysis. Genetic diversity in the BLS regions is elevated, even when NBS-LRR genes themselves have been removed (Additional file 1: Figure S9). Removing *π*-islands with lower coverage depth from BLS regions also did not change our conclusions (Additional file 1: Table S9). These results suggest that the high variation was not due to the read-mapping errors in paralogous loci or highly polymorphic regions. Rather, historical balancing selection could act on NBS-LRR genes and maintain ancestral haplotypes with similar frequency in different geographical lineages of *B. stricta*. The high diversity in the BLS regions after excluding NBS-LRR genes suggested that intergenic regions linked to these genes were affected by selection. The average length of affected haplotypes is ~ 72 kb (20 Mb divided by 277 genes), much longer than the level of LD in this species (Additional file 1: Figure S5). Future studies, taking advantage of long-read sequencing technologies and functional genomic analyses, can reconstruct the ancient haplotypes and examine candidate selected genes.

An early population genomic study in *A. thaliana* found that regions syntenic to these BLS regions also show enrichment for NBS-LRR genes and elevated nucleotide polymorphism [45] (also see Additional file 1: Figure S10). This suggests that variation in the BLS regions may have been shaped by balancing selection over millions of years since these genera diverged [46].

### Sorting of ancient polymorphisms in divergence islands

**Fig. 4:**
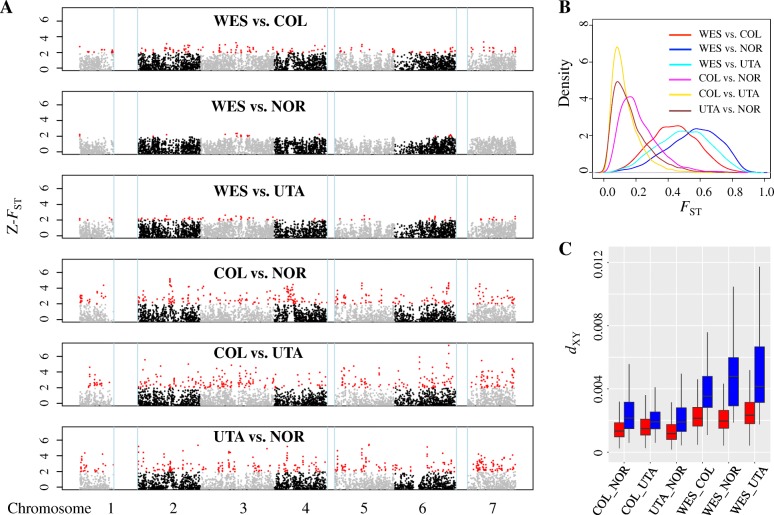
Heterogeneous genetic divergence along the *B. stricta* genome. **a** Manhattan plot of *Z*-transformed *F*_*ST*_ (*Z*-*F*_*ST*_) in 20-kb non-overlapping windows for six group pairs. Alternating colors paint the different chromosomes, and the genomic islands of divergence are shown in red. Windows with low differentiation (*Z*-*F*_*ST*_ < 0) are not shown. The vertical lines (light blue) mark the three highly polymorphic genomic regions (see Fig. 3) that were not included in *F*_*ST*_ analyses. **b**) Kernel distribution of genome-wide differentiation (*F*_*ST*_) for six group pairs. **c** Box plot of absolute divergence (*d*_*XY*_). In each of the six group pairs, mean *d*_*XY*_ values in genomic islands (blue) are significantly higher than those in genomic background (red) (*P* < 10^−5^, randomization test). In these box plots, the median is shown by a horizontal line, while the bottom and top of each box represents the first and third quartiles. The whiskers extend to 1.5 times the interquartile range. Outliers are not shown in the plot

Balanced polymorphisms in ancestral populations could be maintained by long-term balancing selection during speciation, generating genomic regions with increased genetic diversity within and among daughter populations [10]. Also, ancient polymorphisms could be partitioned among descendant lineages, resulting in genomic regions with increased divergence. To look for genomic regions with elevated divergence among groups, we estimated Weir and Cockerham’s weighted *F*_*ST*_ in 20-kb non-overlapping windows across the genome and computed *Z*-transformed *F*_*ST*_ (*Z*-*F*_*ST*_) scores separately in each group pair [3]. Because the BLS regions were under long-term balancing selection, they were excluded from the data analyses in this section. Genomic regions with *Z*-*F*_*ST*_ ≥ 2, corresponding to 3.04% of windows from all comparisons, were identified as outlier windows (“*F*_*ST*_-islands”; Fig. 4). As previously found in other species [3–7], genetic divergence along the genome was highly heterogeneous and *F*_*ST*_-islands scattered across the genome in all group pairs (Fig. 4; Additional file 1: Figure S11). As expected, increased genome-wide differentiation obscures genomic regions with elevated divergence [5]: we detected fewer *F*_*ST*_-islands in comparisons of more divergent groups. For example, more than 300 islands were observed in comparisons among groups of *East* subspecies (e.g., COL, UTA, and NOR, *F*_*ST*_ = 0.12–0.21), but only 22 in a comparison between sympatrically distributed WES and NOR groups (Additional file 1: Table S12), which showed the highest population differentiation (*F*_*ST*_ = 0.56; Additional file 1: Table S3). The coalescent simulation showed that the *F*_*ST*_ values in genomic islands were significantly higher than the simulated results (*W* ranges from 1,188,900 to 35,200,000, *P* < 5e^−16^, Mann-Whitney *U* test; Additional file 1: Figure S12), suggesting that neutral demographic processes cannot explain the elevated differentiation in outlier windows.

#### Relative vs. absolute divergence in divergence islands

To examine which factors have contributed to the formation of *F*_*ST*_-islands [3, 11, 13], for each group pair, we compared the level of *d*_*XY*_ and RND in islands versus the genomic background. We found that both mean *d*_*XY*_ and mean *RND* were significantly higher in *F*_*ST*_-islands of all group pairs (*P* < 10^−5^, randomization test; Fig. 4; Additional file 1: Table S3), suggesting the elevated *d*_*XY*_ in genomic islands was not due to the increased substitution rates. Elevated levels of both relative divergence (*F*_*ST*_) and absolute divergence (*d*_*XY*_) in *F*_*ST*_-islands are compatible with a model in which these islands were derived from divergent sorting of ancient polymorphisms [13, 21]. Consistent with this hypothesis, the coalescent time between the most divergent haplotypes in genomic islands was ~ 1 million years ago, which is much earlier than the split of *B. stricta* groups (Additional file 1: Figure S13). Alternatively, the *F*_*ST*_-islands also could result from differential gene flow among genomic regions, i.e., restricted gene flow in islands versus high gene flow elsewhere in the genome [11]. Under this alternative hypothesis, *F*_*ST*_-islands would be more pronounced in sympatric group pairs, since gene flow could be higher than for allopatric groups. In contrast, *d*_*XY*_ was significantly higher in *F*_*ST*_-islands of both sympatric and allopatric group pairs, and the sympatric group pair (WES and NOR) showed the highest *F*_*ST*_ and the lowest number of *F*_*ST*_-islands among comparisons (Additional file 1: Tables S3 and S12). This indicates low gene flow between *East* and *West* subspecies in their zone of sympatry. Coalescent simulation supported a model with low gene flow among the groups (Fig. 2; Additional file 1: Figure S4; Additional file 1: Table S6), in agreement with the high inbreeding coefficient [17] and low seed dispersal of *Boechera* [15]. The geographically isolated distribution of *B. stricta* groups (except WES and NOR) together with the low frequency of *stricta* × *stricta* admixed genotypes, also suggest that gene flow has been limited among the groups. Therefore, our observations indicate that reduced levels of recent gene flow are unlikely to be an important contributor to the formation of *F*_*ST*_-islands in *B. stricta*.

We further tested whether *F*_*ST*_-islands could be due to the ongoing background selection or recurrent selective sweeps. These two processes tend to reduce genetic polymorphism and to elevate *F*_*ST*_ in low-recombination regions [11], so we compared the recombination rate between *F*_*ST*_-islands and the rest of the genome and tested the correlation between differentiation (*F*_*ST*_ and *d*_*XY*_) and recombination in *F*_*ST*_-islands. Among the 12 comparisons (four group pairs, each containing two tests for two groups), only two comparisons showed a significant lower recombination in *F*_*ST*_-islands than in background (0.668 vs. 1.037, *P* = 0.031; 0.718 vs. 0.901, *P* < 0.001; randomization test with Bonferroni correction; Additional file 1: Table S3). Thus, *F*_*ST*_ values in *F*_*ST*_-islands are significantly higher than background after controlling for recombination and/or gene density (*F*_1,6126–6820_ = 86.43–5123.27, *P* < 2e^−16^; Additional file 1: Table S13). For regression tests, we found two significantly negative relationships out of 12 comparisons. It is possible that other historical factors, such as demographic history, might explain this pattern (Additional file 1: Figure S14). Additionally, if *F*_*ST*_-islands were mainly produced by background selection or recurrent selective sweeps, *d*_*XY*_ within islands would be decreased or unaffected [3, 11], which is inconsistent with our observation of elevated *d*_*XY*_ in genomic islands.

#### Positive selection in divergence islands

Within groups, *F*_*ST*_-islands showed low nucleotide diversity (*π*), excess of low frequency alleles (more negative Tajima’s *D*) ,and high-frequency derived alleles (more negative Fay and Wu’s *H*) in one or both groups of each comparison (*P* < 10^−5^, randomization test; see details in Additional file 1: Table S3), consistent with positive selection in these regions. It has been found that ancient polymorphisms under long-term balancing selection also could be under recent positive selection and promote adaption of humans to local environments [47]. To further infer possible functional influences, we conducted Gene Ontology (GO) analyses of these genomic islands. Comparisons showed that multiple GO categories with important metabolic processes and molecular functions (e.g., sucrose metabolic processes, catalytic activity) were overrepresented for genes located in genomic islands (Additional file 1: Table S14), suggesting a diverse set of genes and functional categories may have contributed to adaptive evolution of *B. stricta*.

## Conclusions

We used population genomic analyses to track the divergence processes of *B. stricta* and to investigate the evolutionary forces that have shaped diversity within this species. We found that four genetic groups in *B. stricta* diverged during the Late Pleistocene. Our results suggest that long-term balancing selection on disease resistance genes may have maintained ancestral haplotypes across descendent lineages, resulting in elevated genetic diversity in three genomic regions comprising 10% of the genome. We further demonstrate that genomic regions with elevated divergence (*F*_*ST*_-islands) among the four lineages may be derived from divergent sorting of ancient polymorphisms, instead of heterogeneous gene flow or recurrent selective sweeps. These findings provide evidence that elevated genetic diversity due to balancing selection also may increase population differentiation by sorting balanced polymorphisms during divergence processes. This study highlights the importance of ancestral balanced polymorphisms as crucial constituents of genome-wide variation and incipient speciation.

## Methods

### Sample collection, whole-genome sequencing, mapping, and SNP calling

We analyzed 517 *B. stricta* accessions across the species range in the western USA (Fig. 1; Additional file 1: Table S1). These accessions are part of the *B. stricta* Reference Panel, available from the *Arabidopsis* Biological Resource Center. All accessions have the common, non-inverted haplotype on chromosome 1 [21]. Seeds were germinated and grown in the greenhouse for one generation to produce self-pollinated seeds for this study. Following the protocol of Lee et al. [21], we extracted genomic DNA of each accession from ~ 0.1 g young leaf tissues using Qiagen DNeasy Plant Mini kits (Qiagen, Hilden, Germany) and measured the concentration using a Qubit fluorometer (Invitrogen, Carlsbad, CA, USA).

Paired-end sequencing libraries were prepared for each sample, and the sequencing was carried out on the Illumina HiSeq 2000/2500 platform at the Joint Genome Institute (JGI). Plate-based DNA library preparation for Illumina sequencing was performed on the PerkinElmer Sciclone NGS robotic liquid handling system using KAPA Biosystems Library Preparation Kit. About 200 ng DNA was sheared to 475–600 bp using a Covaris LE220 Focused-ultrasonicator. The sheared DNA fragments were size selected by double-SPRI, and then the selected fragments were end-repaired, A-tailed, and ligated with Illumina compatible sequencing adaptors from IDT containing a unique molecular index barcode for each sample library. The prepared libraries were quantified using KAPA Biosystem’s next-generation sequencing library qPCR kit and run on a Roche LightCycler 480 real-time PCR instrument. The quantified libraries were then multiplexed with other libraries, and the pool of libraries was then prepared for sequencing on the Illumina HiSeq sequencing platform utilizing a TruSeq paired-end cluster kit and Illumina’s cBot instrument to generate a clustered flowcell for sequencing. Sequencing of the flowcell was performed on the Illumina HiSeq2000/2500 sequencer using a TruSeq SBS sequencing kit, following a 2 × 100 or 2 × 150 indexed run recipe.

We used trimmomatic v0.36 [48] to remove adapter sequences and trim bases from both ends of reads when the base quality was < 30. After trimming, reads < 36 bp were discarded. We then aligned high-quality reads of each genotype to the *B. stricta* reference genome v1.2 [21] with BWA [49]. We used GATK v3.8 [25] for base quality recalibration, indel realignment, and simultaneous SNP and indel discovery via HaplotypeCaller. After that, genotypes (in gVCF files) of all individuals were joined together by using the default hard filtering parameters as prescribed by GATK v3.8 best practices. Only sites with mapping quality ≥ 30 and base quality ≥ 30 were considered for calling variants in HaplotypeCaller.

### SNP filtering and validation

As some analyses required called genotypes, we used stringent filtering criteria to minimize false positives from SNP and genotype calls and further validated the accuracy of variant calling. We used the following filtering criteria: (1) homozygous genotypes were assigned as missing if supported by less than two reads; (2) heterozygous genotypes were assigned as missing if supported by less than 20 reads or reference ratio (number of reads supporting reference allele/number of reads supporting alternative allele) < 0.25, or > 0.75; and (3) SNPs were discarded if they met any one of the following criteria: genotyped in fewer than 50% of individuals, mean depth > 20, more than one variant allele was observed, sites where the proportion of heterozygous genotypes was > 15% (*B. stricta* is predominantly inbred; hence, high heterozygosity may indicate paralogous loci), or if reference or variant alleles were indels. We further removed 20 samples with missing rate > 0.60, five duplicated samples from the same inbred family, six samples with divergent morphology or identified as outliers by PCA, and two samples without geographical information (Additional file 1: Table S1). Finally, we retained 4,125,395 high-quality SNPs and 484 genotypes for the following analyses.

To gain insight into the variant calling and genotype accuracy, we compared individual genotypes from this re-sequencing dataset with genotypes from 129 loci (total 71 kb) previously assayed by Sanger sequencing [18, 50]. Twenty-one inbred lines sequenced by both Illumina and Sanger methods were used for this comparison (Additional file 1: Table S2). Each genotype of 21 individuals (sequenced at depth 2.7–5.8×) inferred by GATK was compared to the Sanger-sequenced ones from the same inbred lines. Genotypes from the re-sequencing data identical to the genotypes from the Sanger dataset were considered as true positives, and conflicting genotypes were considered as false. 99.89% of genotypes from the re-sequencing data are identical to genotypes from the Sanger dataset, indicating that high confidence SNPs were genotyped in our dataset.

To further monitor the accuracy of genotypes over different coverage depths, we downloaded reads of two genotypes that were sequenced at very high depth, ~ 400× (Accession LTM, the genotype used for the reference genome) and ~ 170× (SAD12, referred to as RP067 in this study) [21]. Next, we randomly downsampled these datasets to sub-datasets with depth from 1 to 40× (Additional file 1: Table S15). SNPs were called and filtered from these sub-datasets using the same pipelines as described above. For LTM, we compared genotype calls from sub-datasets with different depths to the reference genome. True positives (TP) are positions identical to the reference, false heterozygotes (FHET) are positions called as heterozygous genotypes (all sites are called as homozygotes in the reference genome), false homozygotes (FHOM) are positions called as homozygous genotypes but different from the reference, and missing SNPs (MISS) are non-genotyped sites. For SAD12, we compared genotypes called from each sub-dataset to those called from the highest depth data (~ 170×). True positive sites (TP) are defined as positions identical between low- and high-depth datasets; false heterozygotes (FHET) are positions different between datasets, with at least one heterozygote called; false homozygotes (FHOM) are positions called as different homozygotes between datasets; and missing SNPs (MISS) are sites genotyped in high-depth data but not called in low-depth data. For both LTM and SAD12, the true positive rate (TPR) is defined as TP/(TP + FHET + FHOM), false discovery rate (FR) is defined as (FHET + FHOM)/(TP + FHET + FHOM), and missing rate (MR) is defined as MISS/(TP + FHET + FHOM + MISS). High TPR was found in LTM (99.90–99.51%) and SAD12 (98.75–99.82%) datasets with different depths (Additional file 1: Table S15). It is not surprising to see high accuracy of called genotypes based on relative low sequencing depth (5.05×) in *B. stricta*, because it is an inbreeding species with extremely low heterozygous rate [17, 21]. A similar result was reported in *A. thaliana*, another inbreeding species [32].

### Population structure

To investigate population structure in *B. stricta*, we (1) conducted admixture analyses using Admixture v1.3.0 [27], FastSTRUCTURE v1.0 [28], sNMF [29], and NGSadmix [30]; (2) performed principal component analysis (PCA) with EIGENSOFT v6.0 [51]; and (3) constructed neighbor-joining (NJ) trees using MEGA v7 [52] with 1000 bootstrap samples. We ran Admixture, FastSTRUCTURE, sNMF, and NGSadmix with *K* values ranging from 1 to 10 and repeated the process 20 times with different seeds. The best *K* (i.e., the number of putative populations) was chosen by the Δ*K* method [53]. A tenfold cross-validation procedure and a cross-entropy criterion were also used for evaluating the runs with different *K* values in Admixture and sNMF, respectively. Genotype likelihoods estimated by ANGSD [26] were used as input for NGSadmix, and genotypes called by GATK were used for other methods. For population structure analyses, we discarded SNPs with missing rate > 20% and minor allele frequency (MAF) < 5%. We also excluded highly correlated SNPs by performing an LD-based SNP pruning process in PLINK v1.90 [54]. To do this, we scanned the genome with sliding windows of 50 SNPs in size, advancing in steps of five SNPs, and removed any SNP with a correlation coefficient > 0.2 with any other SNP within the window. This yielded 27,765 independent SNPs for the analyses of population structure.

### Isolation by distance

To investigate the pattern of isolation by distance in each group, we calculated genetic and geographic distances between each pair of genotypes and tested the correlation of genetic and geographic matrices by Mantel test with 10,000 permutations (permuting rows and columns) implemented in the R package VEGAN [55]. We further quantified the strength of the IBD in each group. To account for uneven sampling, we grouped genotype pairs into sequential 10 km bins (e.g., 0–10 km, 10–20 km) and calculated mean genetic distance from all genotype pairs in each bin. After that, we fit a weighted linear regression by considering the number of genotype pairs in each bin and calculated the slope and intercept of genetic distance against geographic distance. We discarded bins representing geographic distance less than 20 km or larger than 120 km, due to a substantial deviation from the regression line. We also removed bins with less than 15 genotype pairs. The ratio of increase of pairwise diversity across geographic distance was estimated by dividing slope by mean pairwise diversity across all bins within 20–120 km. We estimated the standard error of the ratio based on 1000 bootstraps. We also tried 20 km bins and got very similar results (data not shown). Ten isolated WES accessions from the Washington Cascades and the Sierra Nevada were excluded from IBD analyses, because they are located far from the distribution center of the WES group.

### Linkage disequilibrium

We estimated genome-wide LD species-wide (484 individuals), as well as for each group (WES, COL, UTA, and NOR). We extracted common SNPs with MAF above 0.05 and calculated the mean-squared correlation (*r*^2^) for each pair of common SNPs within 50-kb windows using plink v1.90 [54]. The decay of LD with physical distance (bp) was estimated using nonlinear regression using Eq. 1 of Hill and Weir [56].

### Population demography

#### Fastsimcoal2 simulation

We inferred the demographic history of *B. stricta* by using a coalescent simulation-based method in fastsimcoal v.2.6.0.3 [33]. We tested fourteen demographic models (Additional file 1: Figure S3); all models contained four contemporary groups and began with the splitting of the two subspecies (WES vs. the ancestor of other three groups), followed by splitting of COL within the *East* subspecies, and the final split between UTA and NOR groups. These models differed with regard to (1) whether gene flow was present among groups and (2) how population size changed within groups (Additional file 1: Figure S3). Because missing data can lead to biased estimates of the site frequency spectrum (SFS), we performed a downsampling procedure following Thome and Carstens [57]. For each individual, we randomly chose one haplotype, since *B. stricta* is a largely inbreeding species. For each site, we resampled (without replacement) 39, 78, 63, and 52 genotypes from WES, COL, UTA, and NOR groups, respectively, to maximize the number of segregating SNPs. Sites were discarded if the sample size (non-missing genotypes) was less than the threshold in any group. We excluded SNPs from three genomic regions under long-term balancing selection (see the “Balancing selection in *B. stricta* genomes” section) and only used fourfold degenerate sites and intergenic regions, because they are less affected by selection. Finally, 1,455,094 SNPs were retained to estimate SFS. To minimize biases when determining the ancestral allelic states, we generated folded SFS following the methods described by Excoffier et al. [33]. For each model, we performed 50 independent runs with 100,000 coalescent simulations as well as 10–40 conditional maximization algorithm cycles to find the global maximum-likelihood parameter estimates. The best model was chosen based on Akaike’s weight of evidence following Excoffier et al. [33]. To obtain the 95% confidence interval of the best model, we generated 100 parametric bootstraps and estimated the parameters on each bootstrap replicate using the same settings as for the analyses of the original dataset. Generation time of 2 years and mutation rate of 7 × 10^−9^ substitutions per site per year in *A. thaliana* [34] were used to convert the model parameters to absolute values. To evaluate the goodness-of-fit of demographic models, we performed 100,000 coalescent simulations under the maximum likelihood estimates of population parameters, calculated expected SFS, and compared with the observed SFS. We also compared two summary statistics (*π* and *F*_*ST*_) between simulated and observed data.

#### Stairway plot analyses

We applied the Stairway plot v2 [35] method with folded SFS (generated as for fastsimcoal2, above) to infer the historical changes of *N*_e_ over time in each genetic group. We used default settings to run Stairway, including 2/3 of the data for training and four random break points at (nseq-2)/4, (nseq-2)/2, (nseq-2)*3/4, and nseq-2. As suggested by the authors, we created 200 input files using the provided script and estimated the median and 95% confidence interval of demographic parameters based on these files. We converted estimates to absolute values based on a generation time of 2 years and a mutation rate of 7 × 10^−9^ substitutions per site per year.

### Genome-wide scans for regions with elevated diversity and/or divergence

We partitioned scaffolds into 20-kb windows, and calculated the sequence coverage by counting the number of available sites in each window. To obtain all available sites, we used the “-allSites” argument in GATK and filtered non-segregating sites using the same quality thresholds as for segregating sites (see the “SNP filtering and validation” section). For a window to be included in the downstream analyses, we required (1) at least 5000 sites left, after filtering steps; (2) at least 20 SNPs, for summary statistics based on segregating sites (e.g., Tajima’s *D* and *F*_*ST*_); and (3) at least 2000 bases available from both ingroup and outgroup species, for outgroup statistics requiring outgroup information, such as Fay and Wu’s *H* and RND.

#### Intra-population summary statistics

For each group of *B. stricta* and the species as a whole, we estimated SFS and related population genetic statistics using a probabilistic method implemented in ANGSD v0.919 [26]. We filtered the data by (1) removing reads with a minimal mapping quality of 30 and bases with a minimal quality score of 30 (-minMapQ and -minQ), (2) removing sites with information from less than 50% of individuals (-minInd), (3) removing sites with a *P* value higher than 1 × 10^−4^ (-snp_pval), (4) assigning genotypes as missing if the depth was less than two for an individual, and (5) removing sites that did not pass filtering criteria above (see the “SNP filtering and validation” section). We estimated per-individual inbreeding coefficients in ngsF-HMM [58] and incorporated them into the calculation of SFS in ANGSD. Using genotype likelihoods based on the GATK genotyping model [59], we estimated folded and unfolded SFS and derived a set of population genetic summary statistics in 20-kb windows. We estimated nucleotide diversity (*π*) and Tajima’s *D* on the basis of folded SFS and calculated Fay and Wu’s *H* from the unfolded SFS. We used *B. retrofracta* [21, 36] as the outgroup species to infer the ancestral allelic state to estimate the unfolded SFS.

#### Inter-population summary statistics

We used custom Python scripts to calculate the relative genetic differentiation (Weir and Cockerham’s weighted *F*_*ST*_) [60], absolute divergence (*d*_*XY*_) [61], and net pairwise nucleotide divergence (*d*_*a*_) [61] for six pairwise comparisons among the four genetic groups (Fig. 1). To account for the variable mutation rate across the genome, we also estimated the relative node depth (RND) [39] by dividing *d*_*XY*_ of each group pair with their mean divergence to an outgroup species (*B. retrofracta*) [21, 36]. In each pairwise comparison, parameters were estimated on sites with at least 50% of individuals successfully genotyped per population. For *F*_*ST*_, we calculated two variance components (the numerator and denominator) for each segregating site, averaged them separately, and obtained the window-based estimates as a “ratio of average” [60]. For *d*_*XY*_ and RND, we obtained window-based values by averaging per-site estimates across all sites (both variable and monomorphic) passing the initial quality filters for each window.

#### Recombination rate and gene density

Population-scaled recombination rates (*ρ* = 4*N*_e_c) were estimated for each group using the program LDhelmet v.1.10 [37]. We ran LDhelmet with default parameters (100,000 burn-in iterations, 1000,000 Markov chain iterations, and a block plenty of 50) to estimate recombination rate between each pair of SNPs and then weight-averaged over each 20-kb window. We only used SNPs with MAF > 5% to minimize the effects of rare variants, and only retained windows with at least 10 SNPs left after filtering. Using lookup tables with *θ* = 0.001 (close to estimates of genomic background) and *θ* = 0.01 (close to estimates in three genomic region with elevated diversity) yielded quantitatively identical results (Pearson’s correlation coefficient *r* = 0.999, *P* < 2.2e^−16^); thus, only the results based on *θ* = 0.001 were reported. To account for the influence of effective population size on estimated *ρ*, we divided *ρ* by diversity (*π*) in each 20-kb window following Wang et al. [4] and compared *ρ*/*π* between islands and the rest of the genome. Gene density was estimated as the total length of coding sequences within each of the 20-kb windows.

#### Outlier screen

To identify the genome regions with elevated diversity (*π*), we standardized *π* in each group for each window and identified high-diversity windows with *Z*-*π* ≥ 2 (*π*-island, corresponding to 4.30% of windows from all comparisons). Following the same procedure, we standardized per-window *F*_*ST*_ in each pair of groups to a *Z*-score based on the formula *Z*-*F*_*ST*_ = (*F*_*ST*_ × *F*_*ST*_′)/std-*F*_*ST*_ [3], where *F*_*ST*_ is a per-window estimate, and *F*_*ST*_′ and std-*F*_*ST*_ are the mean and standard deviation of *F*_*ST*_ across windows. We excluded the BLS regions from the analyses because they show evidence of long-term balancing selection and defined windows with *Z*-*F*_*ST*_ ≥ 2 (corresponding to 3.04% of windows from all comparisons) as outliers (*F*_*ST*_-island). Based on 100,000 permutations, we compared genomic islands vs. genome-wide background for possible differences in multiple summary statistics, including recombination rate, diversity, and *F*_*ST*_.

To test whether *π*-islands or *F*_*ST*_-islands could be due to demographic processes solely, we first simulated 100,000 segments (20 kb each, the same size as the windows used to scan the genome) using demographic parameters of the best model estimated by fastsimcoal2 (see the “Population demography” section for details). We performed simulations with different levels of recombination (0, 1, 5, 10, 30, 50, 100, and 250 cM/Mb) covering a wide range of recombination rates in plants [62], and generated eight datasets in total. For each dataset, we estimated *π* and *F*_*ST*_ for groups and group pairs, respectively. Finally, we compared *π* (for *π*-islands) and *F*_*ST*_ (for *F*_*ST*_-islands) in islands versus those from simulated data by using the Mann-Whitney *U* test.

We further focused on 130 *π*-islands shared among all groups. These islands showed higher diversity and stronger signatures of balancing selection than islands specific to particular group(s). These islands were clustered into three genomic regions. We merged shared islands that were separated by less than 1.5 Mb into single genomic segments and delimited these three highly polymorphic genomic regions to 15.3–24.4 Mb on the short arm of chromosome 1 (LG1p), 0.47–3.4 Mb on the short arm of chromosome 5 (LG5p), and 0.45–5.1 Mb on the short arm of chromosome 7 (LG7p). To look for patterns of their homologous regions in *A. thaliana* genome, we used SyMAP v3.4 [63] to identify the collinear regions between *B. stricta* [21] and *A. thaliana* genomes [32] and further blasted sequences of the three *B. stricta* genomic regions onto the *A. thaliana* genome to delimit the homologous regions. To calculate genetic diversity in *A. thaliana*, we downloaded SNP data from http://1001genomes.org/. Of the 1135 sequenced accessions, we retained 972 non-relicts from the native range [21, 32]. Genotypes supported by less than 2 reads were assigned as missing. SNPs with indels, more than two alleles, more than 50% missing data, or located on masked genomic regions were further excluded. We partitioned *A. thaliana* into 10-kb, 20-kb, and 100-kb windows, calculated per-site nucleotide diversity (*π*) using custom Python scripts, and obtained the window-based values by averaging per-site estimates in each window. To correct for variation in mutation rate among genomic regions, we divided diversity by divergence to the outgroup species, *Arabidopsis lyrata*.

### Gene ontology enrichment analyses

We performed GO analyses to test whether any functional classes of genes were over-represented in *π-*islands or *F*_*ST*_-islands. We first calculated *P* values of Fisher’s test and subsequently corrected *P* values for multiple testing with Benjamini-Hochberg FDR [64]. GO terms with FDR < 0.05 were considered as significantly enriched. GO analyses were conducted with singular enrichment analysis in agriGO’s Term Enrichment tool [65] and used *B. stricta* genome [21] as a reference.

## Additional file

Additional file 1: Supplementary figures and tables. (PDF 8011 kb)

## Data Availability

The short reads of each genotype have been deposited under GenBank accession numbers SRP054739, SRP134356, SRP134462, SRP134522, and SRP134640. All SNPs used in population genetic analyses, locations of all accessions, and custom scripts are available in the Dryad Data Archive at 10.5061/dryad.574pc6n [66]. Seeds from these accessions are available from the Arabidopsis Biological Resource Center.

## Abbreviations

ANOVA: Analyses of variance
BLS: Balancing selection
GO: Gene ontology
KYA: Thousand years ago
LD: Linkage disequilibrium
MAF: Minor allele frequency
NBS-LRR: Nucleotide-binding site-leucine-rich repeat
NJ: Neighbor joining
PCA: Principal component analysis
