# Correction to: Ancient polymorphisms contribute to genome-wide variation by long-term balancing selection and divergent sorting in *Boechera stricta*

**DOI:** 10.1186/s13059-019-1781-5

**Published:** 2019-08-09

**Authors:** Baosheng Wang, Julius P. Mojica, Nadeesha Perera, Cheng-Ruei Lee, John T. Lovell, Aditi Sharma, Catherine Adam, Anna Lipzen, Kerrie Barry, Daniel S. Rokhsar, Jeremy Schmutz, Thomas Mitchell-Olds

**Affiliations:** 1 Key Laboratory of Plant Resources Conservation and Sustainable Utilization, South China Botanical Garden, Chinese Academy of Sciences, Guangzhou, 510650 China; 2 Department of Biology, Duke University, Box 90338, Durham, NC 27708 USA; 3 Institute of Ecology and Evolutionary Biology and Institute of Plant Biology, National Taiwan University, Taipei, 10617 Taiwan, Republic of China; 4 HudsonAlpha Institute for Biotechnology, Huntsville, AL 35806 USA; 5 Department of Energy Joint Genome Institute, Walnut Creek, CA 94598 USA

**Correction to: Genome Biol (2019) 20:126**

**https://doi.org/10.1186/s13059-019-1729-9**

Following publication of the original article [1], the authors reported that the Availability of data and materials section required updating. The updated text reads as follows:

The short reads of each genotype have been deposited under GenBank accession numbers SRP054739, SRP134283-SRP134373, SRP134393-SRP134433, SRP134436-SRP134479, SRP134481-SRP134572 and SRP134581-SRP134671. All SNPs used in population genetic analyses, locations of all accessions, and custom scripts are available in the Dryad Data Archive at 10.5061/dryad.574pc6n [66]. Seeds from these accessions are available from the Arabidopsis Biological Resource Center.
