# Supplementary material for: Ancient polymorphisms contribute to genome-wide variation by long-term balancing selection and divergent sorting in Boechera stricta
Source: Genome Biol. 2019 Jun 21;20:126. doi: 10.1186/s13059-019-1729-9 (PMC6587263; doi:10.1186/s13059-019-1729-9)
Supplement: Supplementary file 1 — Supplementary figures and tables. (PDF 8011 kb) [file 13059_2019_1729_MOESM1_ESM.pdf]

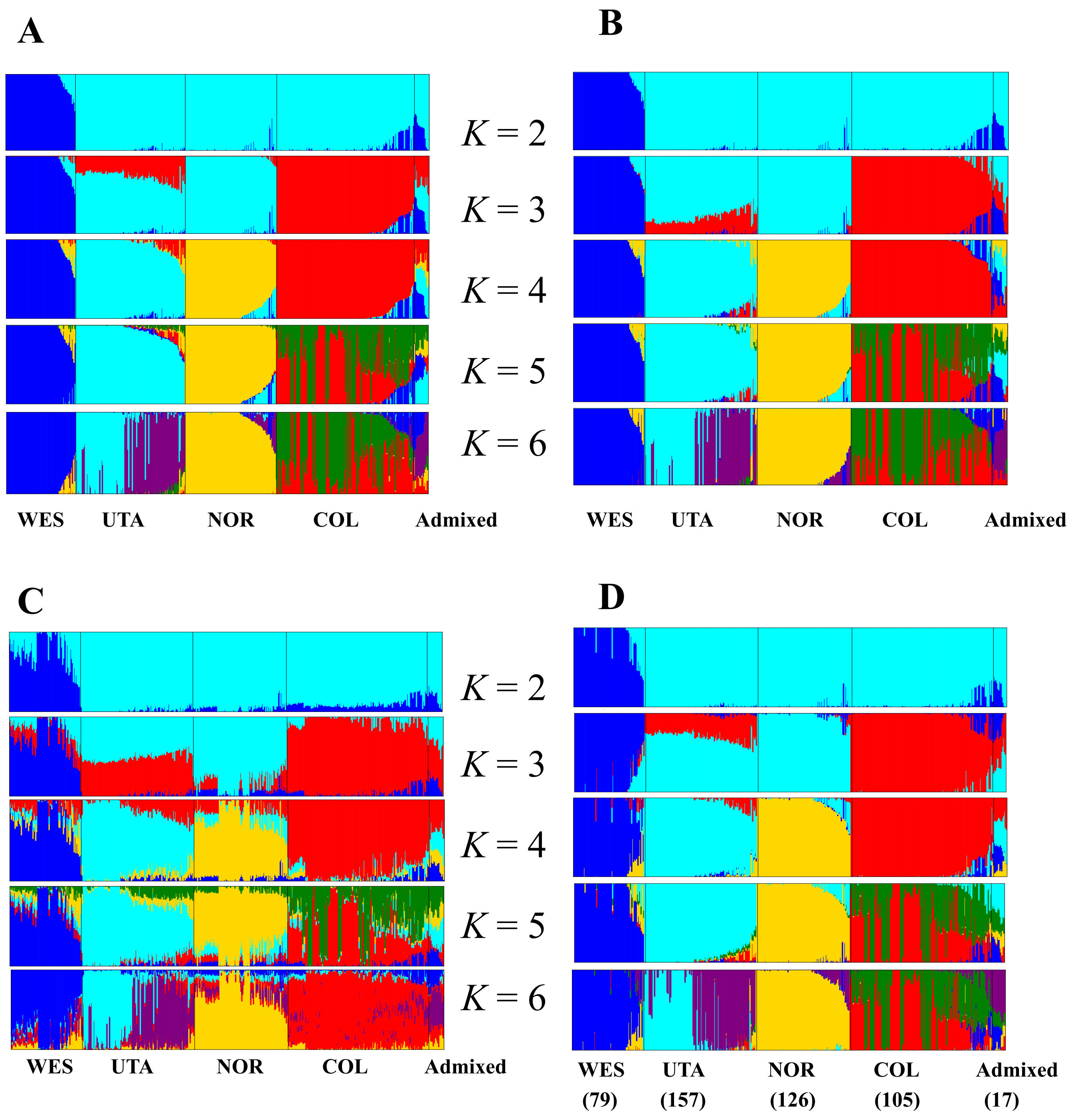

Fig. S1. Admixture (A), FastSTRUCTURE (B), sNMF (C) and NGSadmix (D) analyses from K= 2 − 6 on SNP data for 484 accessions of *B. stricta*. Each vertical bar represents an individual, with different colors representing one of the genetic ancestries. The number of individuals in each lineage is shown in D.

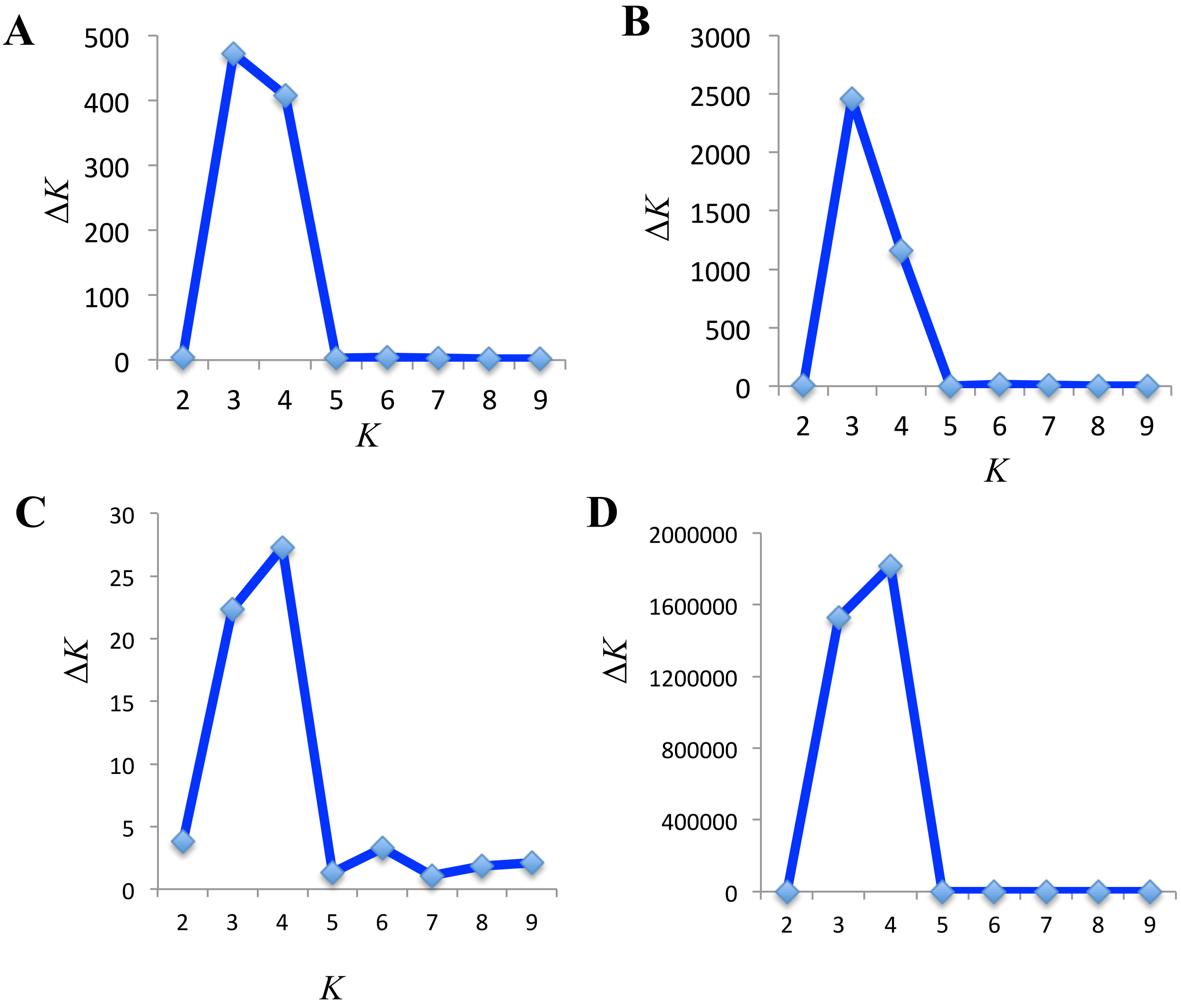

Fig. S2. Detection of the best grouping number (*K*) for the Admixture (A), FastSTRUCTURE (B), sNMF (C) and NGSadmix (D) analyses. Δ*K* values were estimated from Cross-Validation (CV) error in Admixture, marginal likelihood in FastSTRUCTURE (B), Cross-Entropy (CE) criterion in sNMF, and best likelihood in NGSadmix (D). The Δ*K* method evaluates the rate of change of the likelihood (or CV error, CE criterion) function between successive *K* values and identifies the optimal number of clusters as the one giving highest value of Δ*K*. Following this method, we found *K* = 3 and *K* = 4 to be best choices for the number of putative clusters of the 484 individuals.

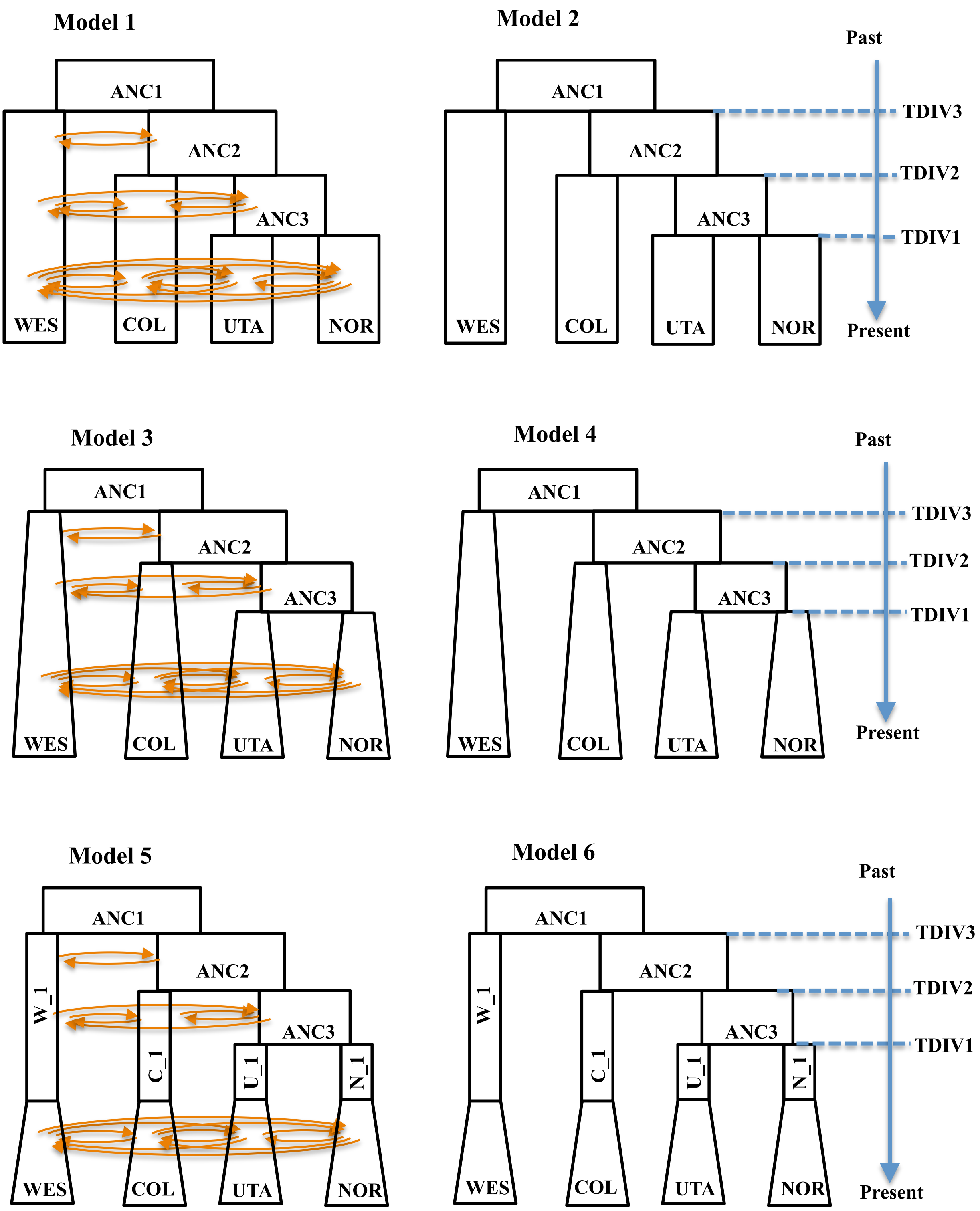
Model 1
ANC1
ANC2
ANC3
WES
COL
UTA
NOR
Model 2
Past
ANC1
ANC2
ANC3
TDIV3
TDIV2
TDIV1
WES
COL
UTA
NOR
Present
Model 3
ANC1
ANC2
ANC3
WES
COL
UTA
NOR
Model 4
Past
ANC1
ANC2
ANC3
TDIV3
TDIV2
TDIV1
WES
COL
UTA
NOR
Present
Model 5
ANC1
ANC2
ANC3
W_1
C_1
U_1
N_1
WES
COL
UTA
NOR
Model 6
Past
ANC1
ANC2
ANC3
TDIV3
TDIV2
TDIV1
W_1
C_1
U_1
N_1
WES
COL
UTA
NOR
Present

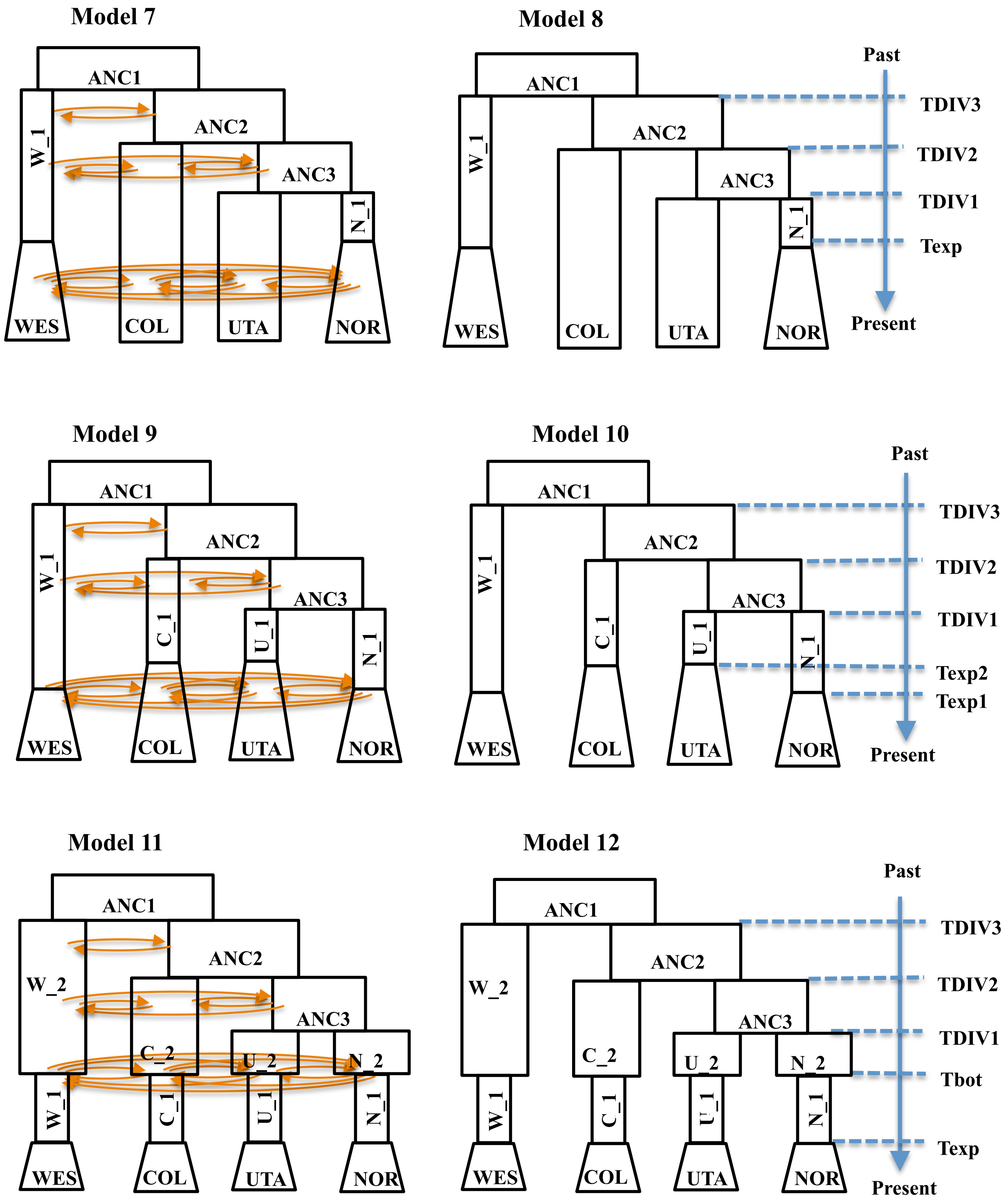

Model 7
ANC1
ANC2
ANC3
W_1
N_1
WES
COL
UTA
NOR
Model 8
ANC1
ANC2
ANC3
W_1
N_1
WES
COL
UTA
NOR
Past
TDIV3
TDIV2
TDIV1
Texp
Present
Model 9
ANC1
ANC2
ANC3
W_1
C_1
U_1
N_1
WES
COL
UTA
NOR
Model 10
ANC1
ANC2
ANC3
W_1
C_1
U_1
N_1
WES
COL
UTA
NOR
Past
TDIV3
TDIV2
TDIV1
Texp2
Texp1
Present
Model 11
ANC1
ANC2
ANC3
W_2
C_2
U_2
N_2
W_1
C_1
U_1
N_1
WES
COL
UTA
NOR
Model 12
ANC1
ANC2
ANC3
W_2
C_2
U_2
N_2
W_1
C_1
U_1
N_1
WES
COL
UTA
NOR
Past
TDIV3
TDIV2
TDIV1
Tbot
Texp
Present

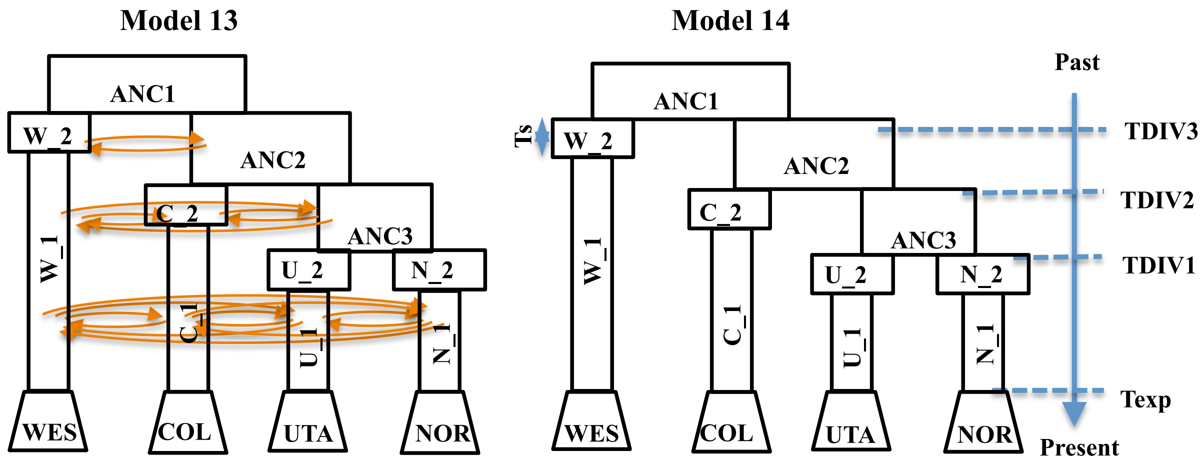

Fig. S3. Fourteen models for isolation-with-gene-flow (Nos. 1, 3, 5, 7, 9, 11 and 13) and isolation-without-gene-flow (Nos. 2, 4, 6, 8, 10, 12 and 14). In models 1 and 2, all groups have constant population sizes after splitting. Models 3 and 4, all groups experienced exponential population size change after splitting. Models 5 and 6, all groups expanded exponentially at a recent time after splitting of group NOR. Models 7 and 8, groups NOR and WES expanded exponentially at a recent time after splitting of group NOR. Groups COL and UTA have constant population sizes after splitting. Models 9 and 10, groups expanded exponentially at different times after splitting of group NOR. Groups UTA and COL expanded earlier than groups NOR and UTA. Models 11 and 12, all groups experienced two steps of population size changes, an instantaneous size change at time T_{bot} and an exponential size change initiated at time $T_{exp.}$ Model 13 and 14, each group experienced a population size change after splitting, within a short period less then 100 generations (T_s). T_s is the same for all groups. Afterwards, all groups experienced a population expansion initiated at time $T_{exp.}$

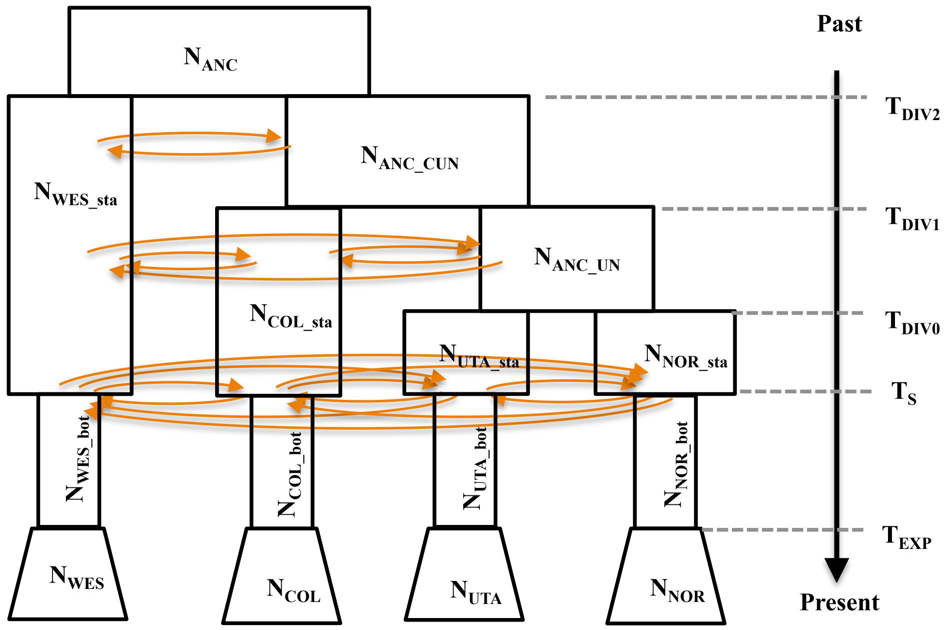

Fig. S4. The best-fit demographic model inferred by *fastsimcoal2*. Parameters with N denote effective population sizes of current and ancestral populations, and with T indicate time estimates of divergence (T_{DIV0}, T_{DIV1} and T_{DIV2}) and population expansion (T_{EXP}). T_S is time of instantaneous size change. The arrows show the migration rate among groups. The inferred demographic parameters are shown in Table S6 and described in the text.

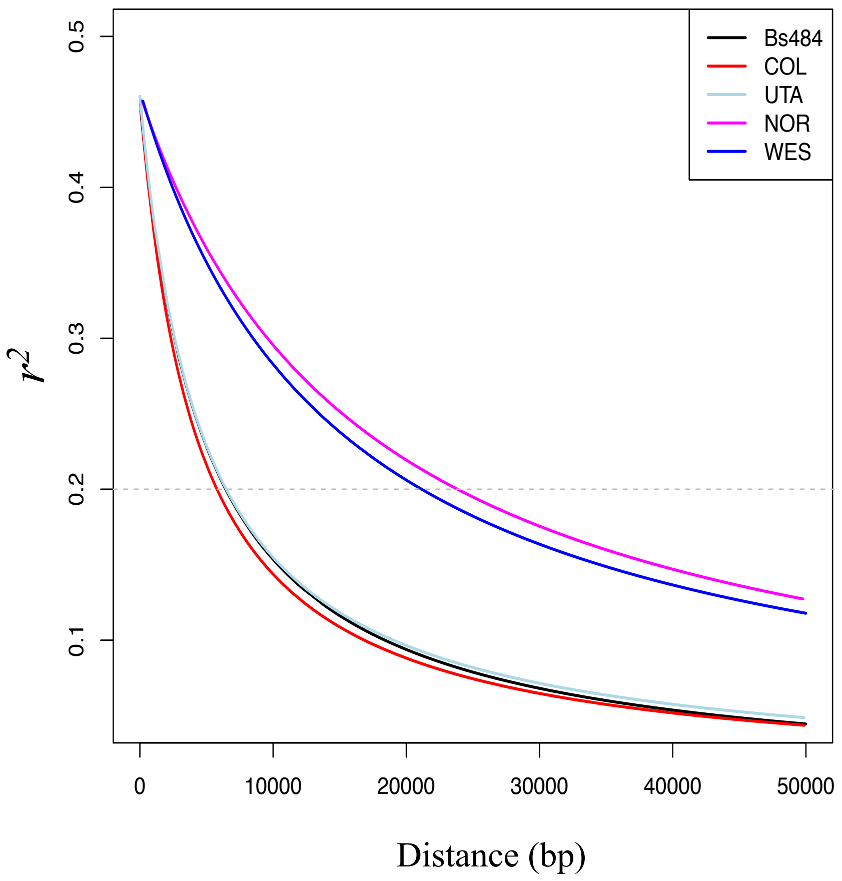

Fig. S5. Genome-wide linkage disequilibrium (LD) in *B. stricta* based on common SNPs (minor allele frequency ≥ 0.05). LD measured by r^2 reaches background levels within ~50 kb in the species-wide sample (Black), COL (Red), and UTA (light blue) groups, but extends longer in NOR (Pink) and WES (Blue) groups.

A

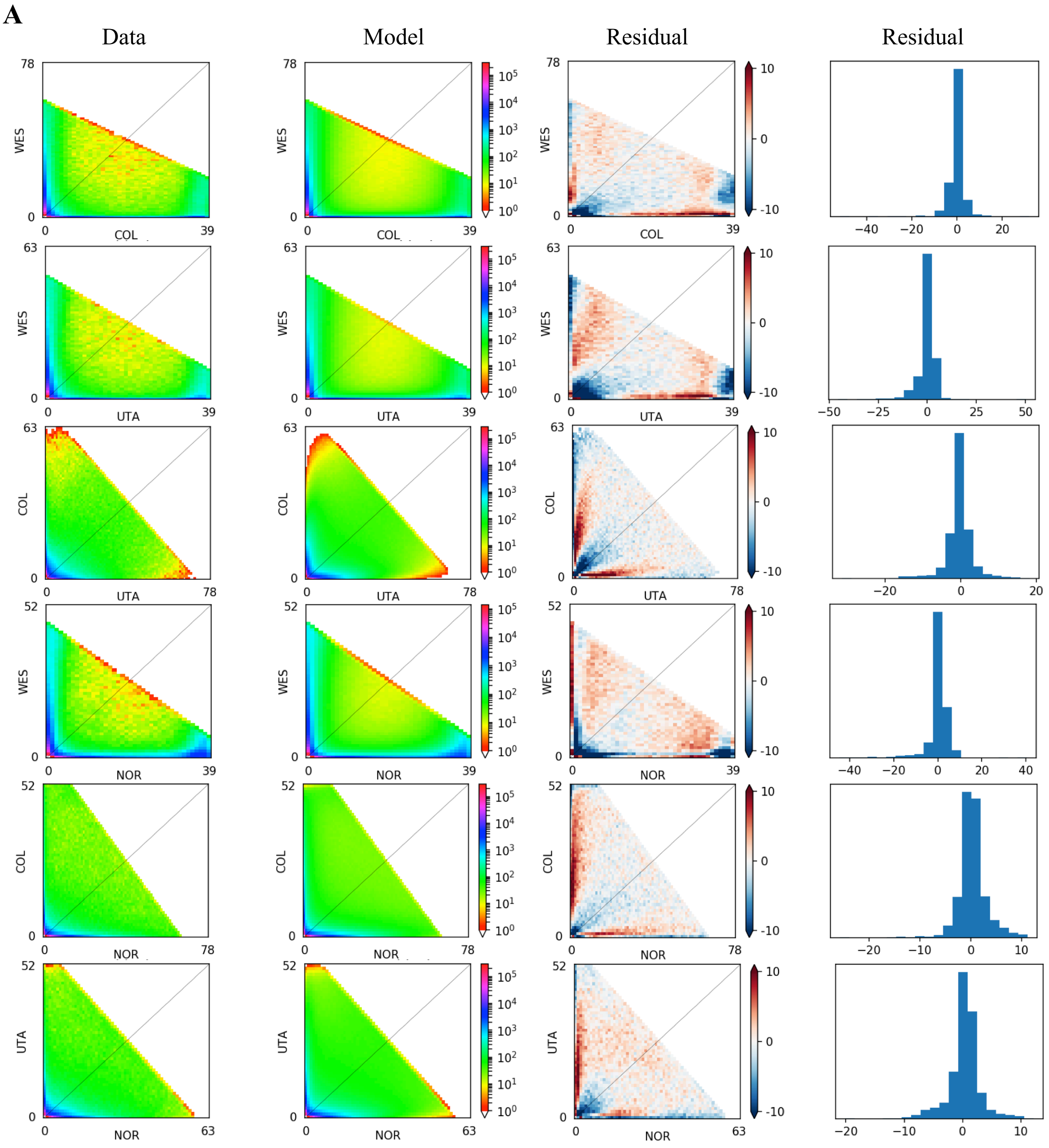

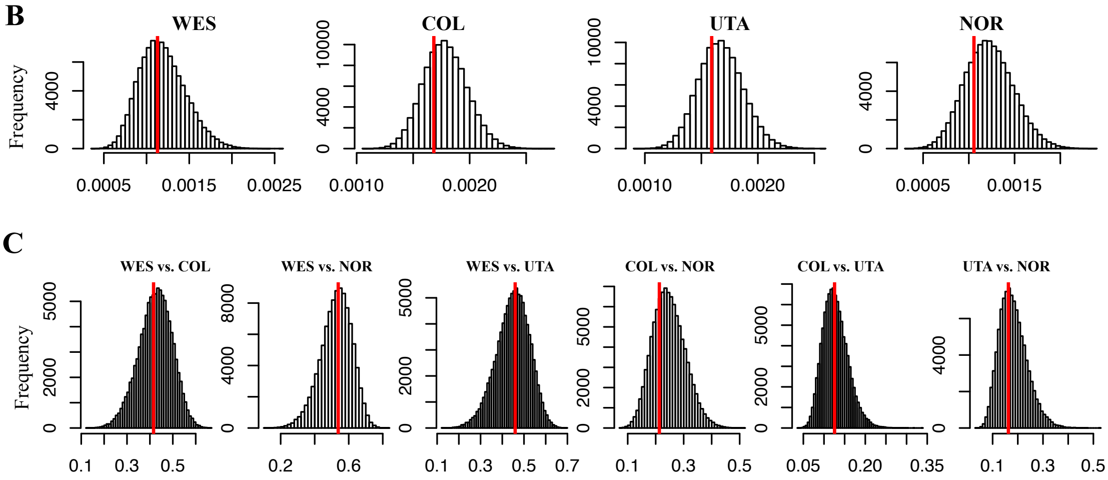

Fig. S6. The goodness-of-fit of the best-fit demographic model inferred by fastsimcoal2. (A), comparison between observed (data) joint site frequency spectrum (SFS) and expected (Model) joint SFS. The residual between Data and Model are plotted in a colormap and a histogram. In the color map, red or blue residuals indicate that model predicts too many or too few alleles in a given cell, respectively. (B), predicted distributions of simulated diversity (π) in each group. (C), predicted distributions of simulated F_{ST} values in each group-pair. Red vertical bars indicate the mean values of observed data in B and C.

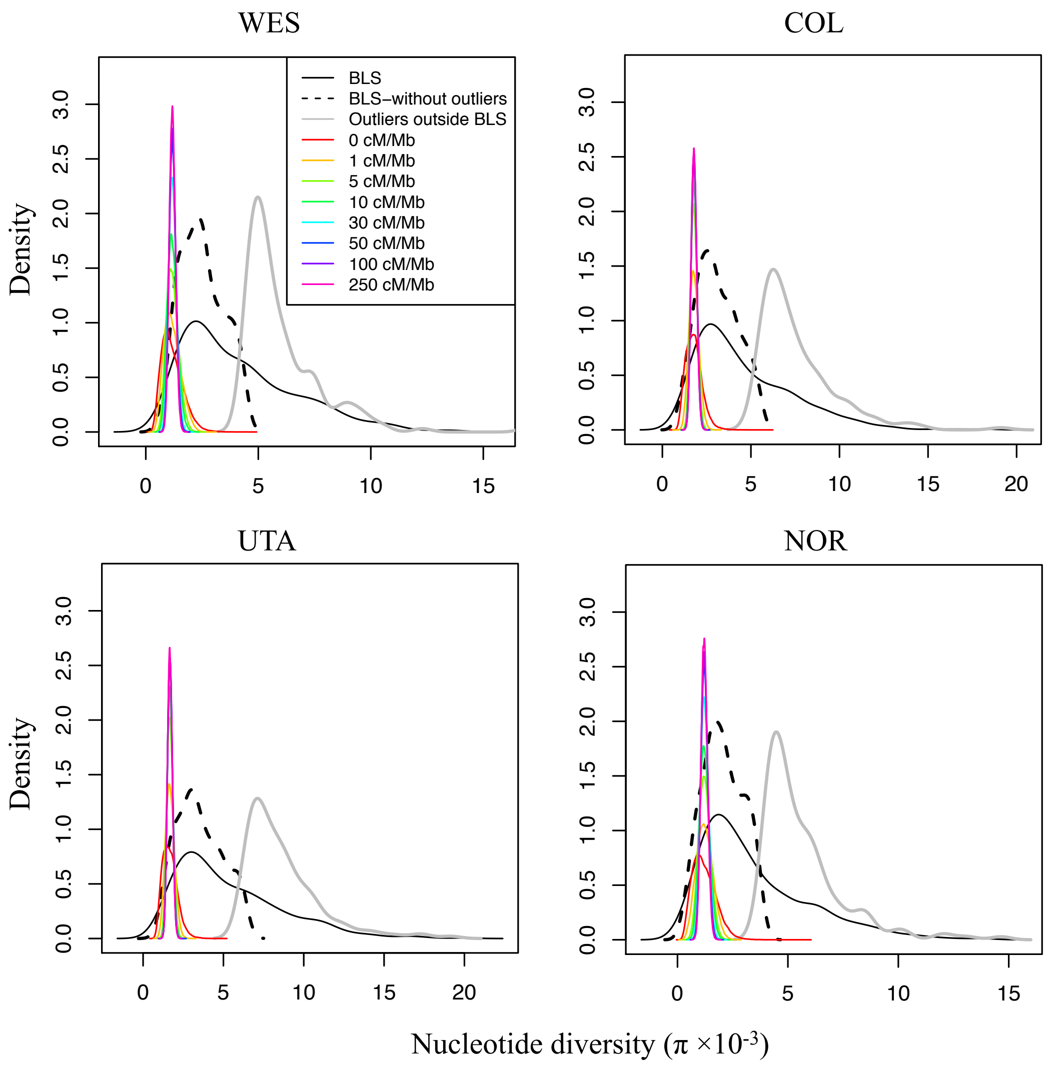

Fig. S7. Predicted distributions of 100,000 simulated diversity (π, in unit of 10^{-3}) values in each group (WES, NOR, COL and UTA) under the best-fit demographic model (see Fig. S4). Simulated results based on different recombination rates are show in colored lines. Black, dashed and grey lines show respectively: observed π values in the BLS genomic regions, BLS regions after excluding π-islands, and genome-wide π-islands outside BLS regions. For these three empirical distributions, the vertical axis is expanded 5-fold for visual clarity. Simulated results are significantly lower than the three empirical distribution (*W* ranges from 8322600 to 45812000, $P < 2e^{-16}$, Mann–Whitney U test).

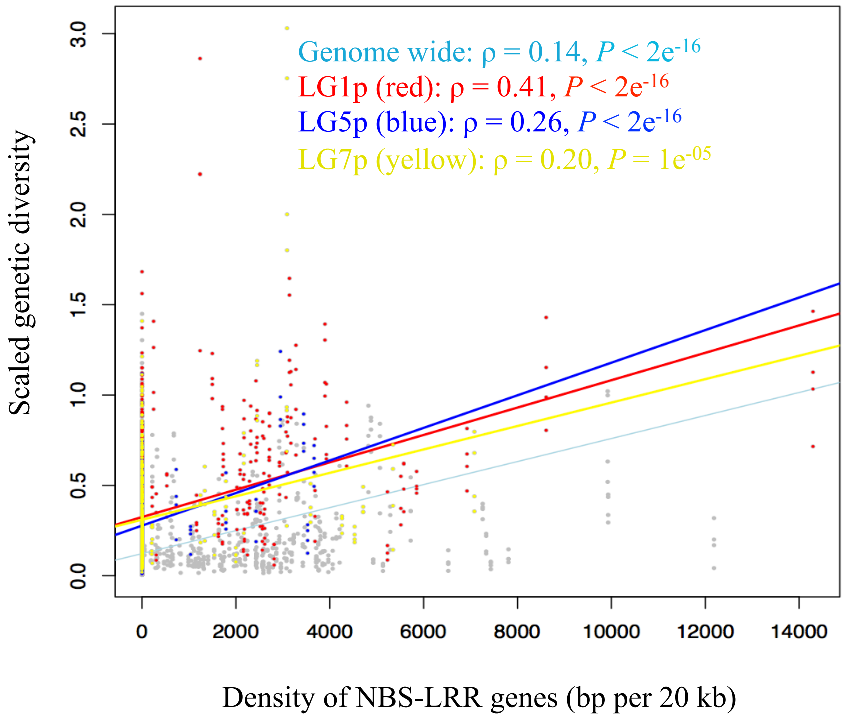

Fig. S8. Correlation between scaled genetic diversity and density of NBS-LRR genes (total length of NBS-LRR genes in a 20-kb window). Correlation and significance are tested with Spearman's rank correlation test in LG1p (red), LG5p (blue), LG7p (yellow) and genome-wide.

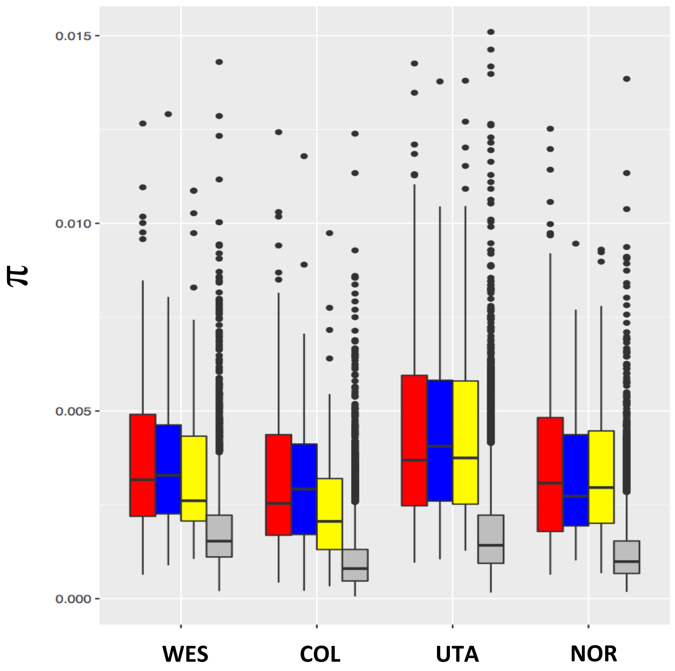

Fig. S9. Comparisons of nucleotide diversity (*π*) in LG1p (red), LG5p (blue), LG7p (yellow) regions versus genomic background (grey), after excluding NBS-LRR gene regions from the three genomic regions. Genetic diversity in the three regions is significantly higher than genomic background in all groups ($P < 10^{-5}$, randomization test).

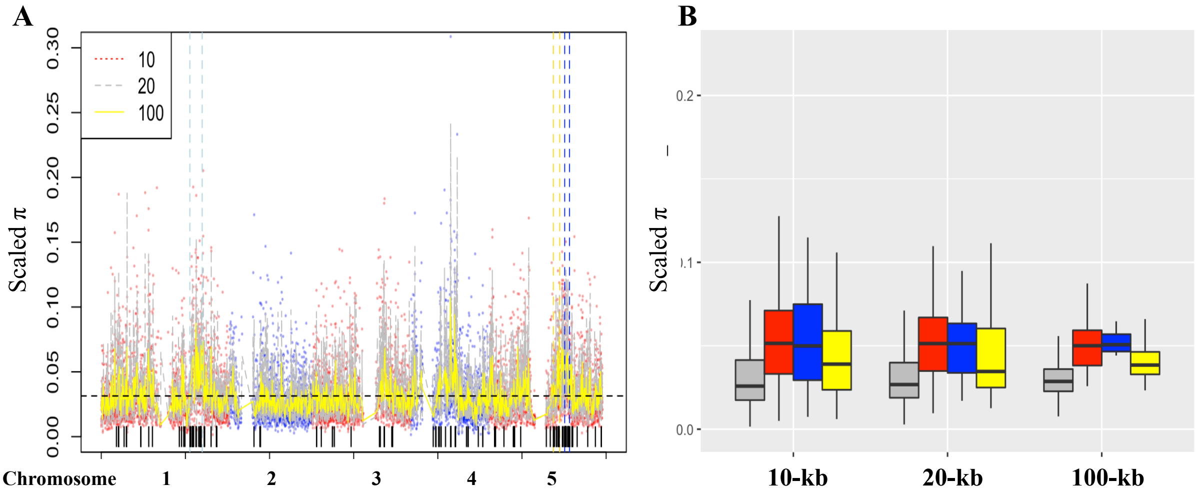

Fig. S10. Genome-wide pattern of nucleotide diversity in *A. thaliana*. (A), Scaled nucleotide diversity (π) is plotted along the genome with windows of 10-kb (red and blue dots for alternating chromosomes), 20-kb (grey dashed line), and 100-kb (yellow line) non-overlapping windows. The dashed vertical lines mark three highly polymorphic genomic regions that are collinear in *B. stricta*. The dashed horizontal line indicates genome-wide average of π across 10-kb windows. NBS-LRR genes are indicated by black bars under the Manhattan plot. (B), Scaled nucleotide diversity (π) in three collinear regions in *B. stricta*, LG1p (red), LG5p (blue) and LG7p (yellow) is significant higher than that in genomic background (grey). $P < 0.01$ for 100-kb windows of LG7p vs. background; $P < 0.001$ for all other comparisons. In each box plot, the median is shown by a horizontal line, while the bottom and top of each box represents the first and third quartiles. The whiskers extend to 1.5 times the interquartile range. Outliers are not shown in the plot.

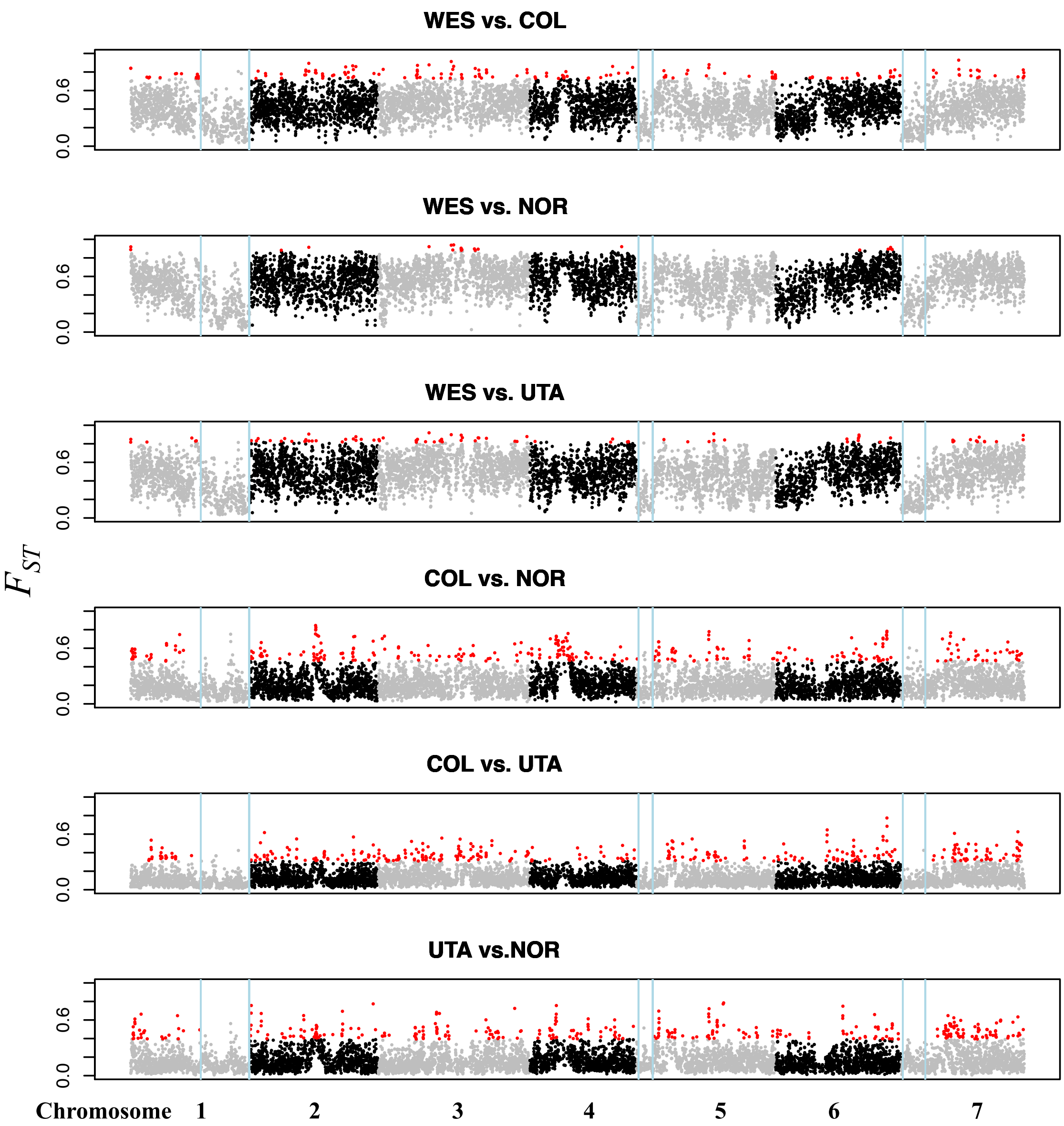

Fig. S11. Manhattan plot of F_{ST} in 20-kb non-overlapping windows for six group pairs. Alternating colors paint the different chromosomes, and the genomic islands of divergence are shown in red. The vertical lines (light blue) mark the three highly polymorphic genomic regions (see Fig. 3) that were not included in F_{ST} analyses.

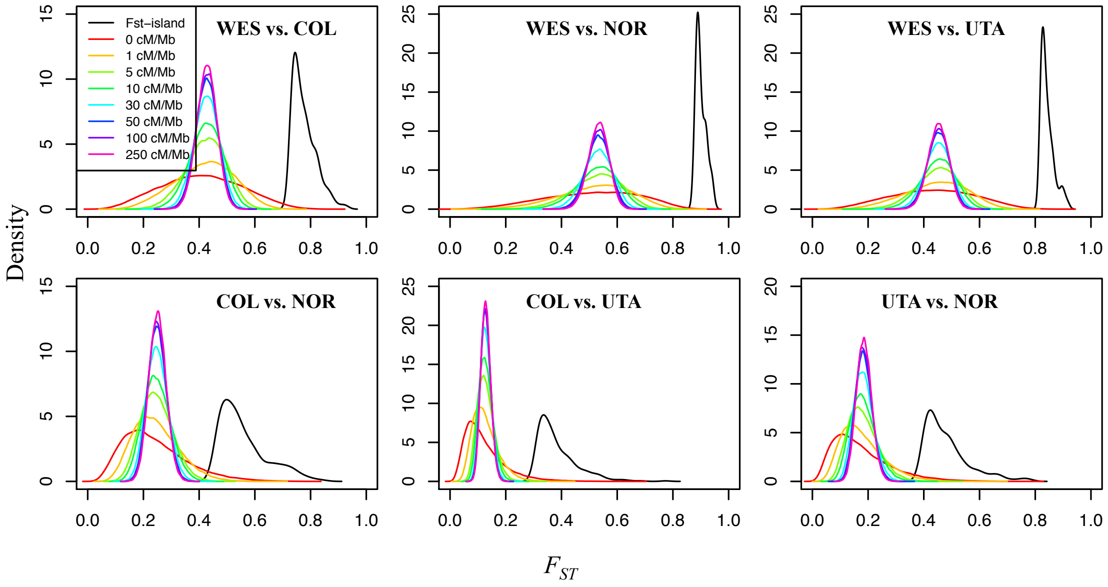

Fig. S12. Predicted distributions of 100,000 simulated F_{ST} values among groups under the best-fit neutral demographic model (see Fig. S4). Simulated results based on different recombination rates (showed in colored lines) are significantly lower (*W* ranges from 1188900 to 35200000, $P < 5e^{-16}$, Mann–Whitney U test) than observed F_{ST} values in F_{ST}–islands (showed in black).

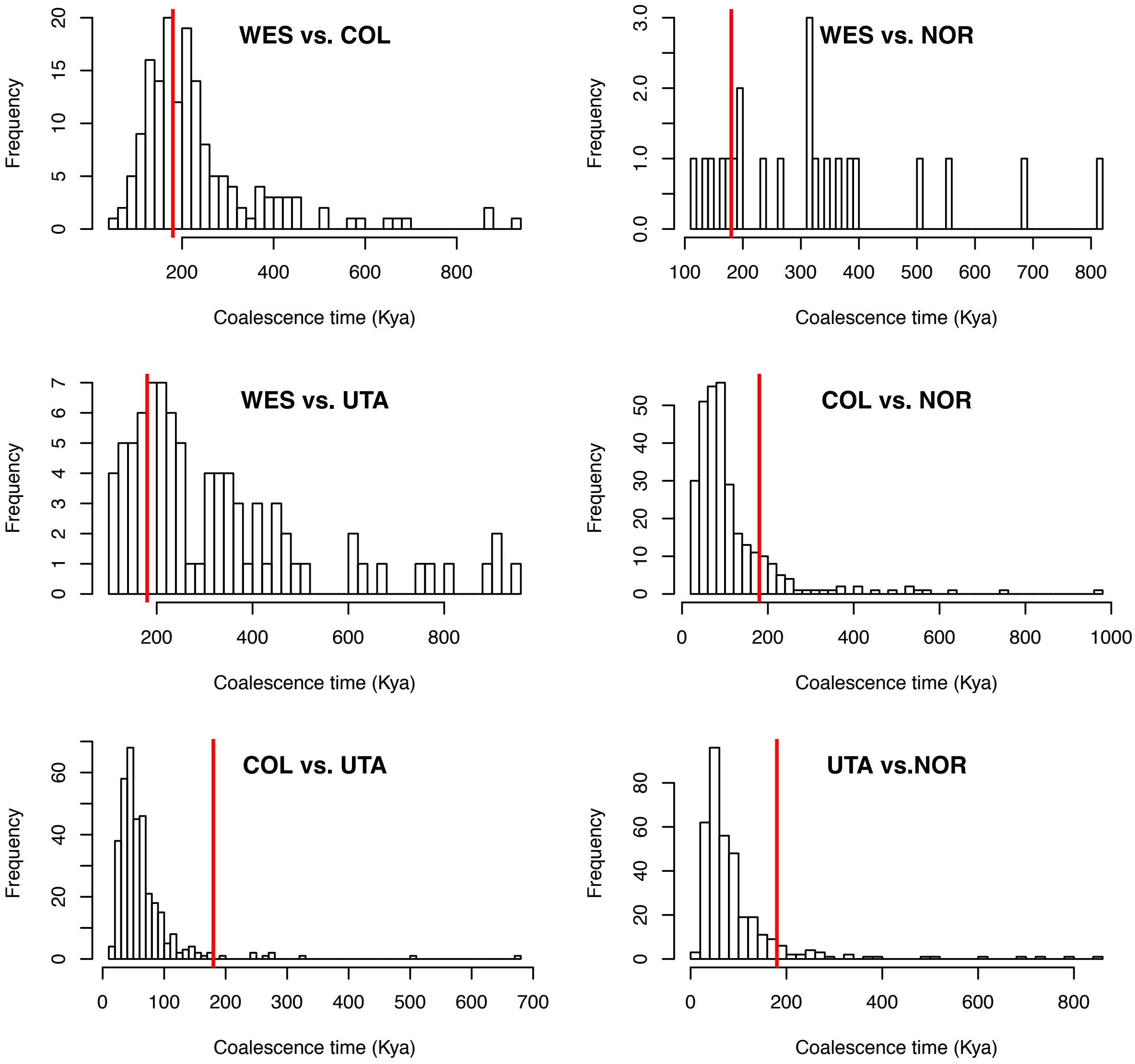

Fig. S13. Coalescence time between haplotypes in 20-kb outlier windows, estimated using $T = D_A/2\mu$, where μ is 7×10^{-9} per site per year. The red vertical line shows the splitting time between *WEST* and *EAST* subspecies of *Boechera stricta*.

A

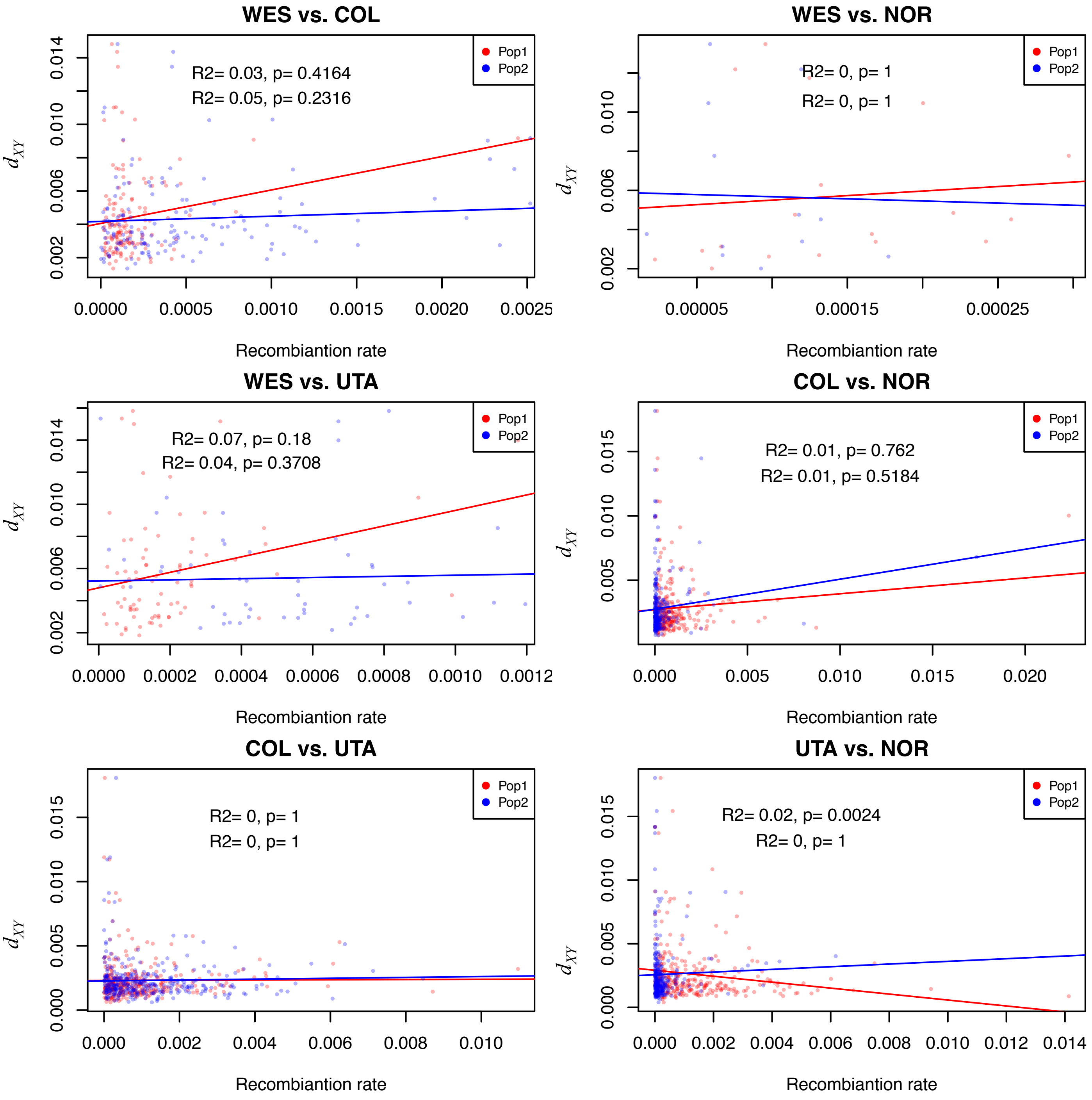

WES vs. COL
R2= 0.03, p= 0.4164
R2= 0.05, p= 0.2316
Pop1
Pop2
d_{XY}
0.002
0.006
0.010
0.014
0.0000
0.0005
0.0010
0.0015
0.0020
0.0025
Recombiantion rate
WES vs. NOR
R2= 0, p= 1
R2= 0, p= 1
Pop1
Pop2
d_{XY}
0.002
0.006
0.010
0.00005
0.00015
0.00025
Recombiantion rate
WES vs. UTA
R2= 0.07, p= 0.18
R2= 0.04, p= 0.3708
Pop1
Pop2
d_{XY}
0.002
0.006
0.010
0.014
0.0000
0.0002
0.0004
0.0006
0.0008
0.0010
0.0012
Recombiantion rate
COL vs. NOR
R2= 0.01, p= 0.762
R2= 0.01, p= 0.5184
Pop1
Pop2
d_{XY}
0.005
0.010
0.015
0.000
0.005
0.010
0.015
0.020
Recombiantion rate
COL vs. UTA
R2= 0, p= 1
R2= 0, p= 1
Pop1
Pop2
d_{XY}
0.000
0.005
0.010
0.015
0.000
0.002
0.004
0.006
0.008
0.010
Recombiantion rate
UTA vs. NOR
R2= 0.02, p= 0.0024
R2= 0, p= 1
Pop1
Pop2
d_{XY}
0.000
0.005
0.010
0.015
0.000
0.002
0.004
0.006
0.008
0.010
0.012
0.014
Recombiantion rate

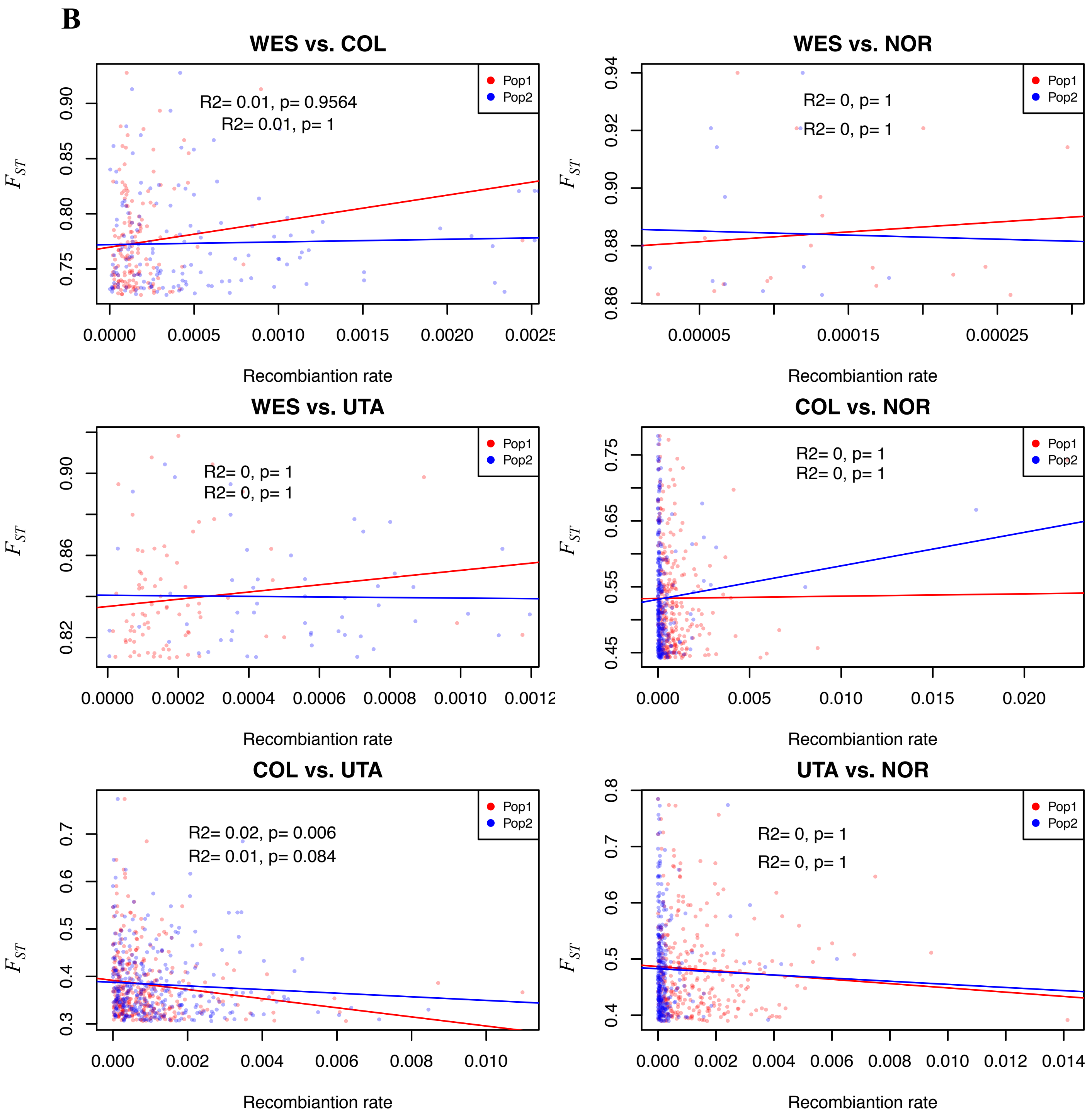

Fig. S14. Relationship between population scaled-recombination rate (ρ = $4N_e c$) and genetic differentiation (d_{XY}: A and F_{ST}: B) for six pairwise combinations of groups. In each plot, adjusted R^2 of linear regression and Bonferroni corrected P-value are shown.

Table S1 Whole genome re-sequenced inbred *Boechera stricta* accessions.

SampleID	Latitude	Longitude	Group	note
RP028	42.499000	-110.794900	Admixed	Included in data analyses
RP044	42.970000	-109.766200	Admixed	Included in data analyses
RP096	42.581000	-110.739600	Admixed	Included in data analyses
RP155	42.506000	-110.678100	Admixed	Included in data analyses
RP176	42.475000	-110.616600	Admixed	Included in data analyses
RP217	43.003000	-109.753900	Admixed	Included in data analyses
RP277	42.673130	-108.876900	Admixed	Included in data analyses
RP304	38.994270	-107.065510	Admixed	Included in data analyses
RP436	45.103070	-111.870870	Admixed	Included in data analyses
RP437	45.025650	-111.846020	Admixed	Included in data analyses
RP440	44.549270	-112.091300	Admixed	Included in data analyses
RP443	44.486200	-112.112270	Admixed	Included in data analyses
RP451	45.085750	-111.864050	Admixed	Included in data analyses
RP452	45.054870	-111.839200	Admixed	Included in data analyses
RP455	44.924870	-111.836230	Admixed	Included in data analyses
RP458	44.892200	-111.655170	Admixed	Included in data analyses
RP460	40.615950	-111.507380	Admixed	Included in data analyses
HET21	39.648880	-107.159130	COL	Included in data analyses
RP001	38.506000	-109.318900	COL	Included in data analyses
RP015	38.945000	-106.988400	COL	Included in data analyses
RP026	40.693000	-109.494600	COL	Included in data analyses
RP029	38.856000	-107.061300	COL	Included in data analyses
RP031	39.761000	-110.919400	COL	Included in data analyses
RP032	40.399630	-106.119000	COL	Included in data analyses
RP034	39.906670	-105.517170	COL	Included in data analyses
RP036	38.988500	-107.006800	COL	Included in data analyses
RP042	40.001000	-105.468100	COL	Included in data analyses
RP051	38.898000	-107.202600	COL	Included in data analyses
RP057	38.692000	-106.822900	COL	Included in data analyses
RP058	40.309000	-106.062100	COL	Included in data analyses
RP065	38.976100	-106.989700	COL	Included in data analyses
RP067	38.707000	-106.804500	COL	Included in data analyses
RP073	39.951000	-105.593600	COL	Included in data analyses
RP077	38.969500	-106.967200	COL	Included in data analyses
RP083	38.775000	-106.626900	COL	Included in data analyses
RP085	38.301000	-107.355800	COL	Included in data analyses
RP086	38.730000	-106.820100	COL	Included in data analyses
RP100	38.510000	-109.318800	COL	Included in data analyses
RP110	38.725700	-106.759400	COL	Included in data analyses
RP111	38.762000	-106.862800	COL	Included in data analyses

RP125	39.684000	-105.607100	COL	Included in data analyses
RP133	40.080000	-105.532500	COL	Included in data analyses
RP139	40.544000	-106.625000	COL	Included in data analyses
RP146	39.000000	-107.022300	COL	Included in data analyses
RP151	38.850000	-107.100900	COL	Included in data analyses
RP152	39.864000	-105.444700	COL	Included in data analyses
RP153	38.543000	-109.301800	COL	Included in data analyses
RP156	38.958000	-106.988800	COL	Included in data analyses
RP164	38.963000	-106.987600	COL	Included in data analyses
RP175	38.976000	-106.998600	COL	Included in data analyses
RP195	37.807600	-109.818700	COL	Included in data analyses
RP199	44.404000	-103.938400	COL	Included in data analyses
RP220	38.666000	-106.838700	COL	Included in data analyses
RP221	38.858000	-107.154700	COL	Included in data analyses
RP229	38.828000	-106.835300	COL	Included in data analyses
RP234	38.963000	-106.992500	COL	Included in data analyses
RP245	38.768000	-106.766000	COL	Included in data analyses
RP255	38.727000	-106.761800	COL	Included in data analyses
RP261	38.746100	-106.673000	COL	Included in data analyses
RP262	38.758000	-106.680300	COL	Included in data analyses
RP265	38.807000	-106.563200	COL	Included in data analyses
RP270	44.300750	-107.305780	COL	Included in data analyses
RP272	40.520600	-105.892700	COL	Included in data analyses
RP273	42.744510	-106.325100	COL	Included in data analyses
RP274	44.282760	-106.991600	COL	Included in data analyses
RP275	41.226670	-105.382920	COL	Included in data analyses
RP278	41.339170	-106.375280	COL	Included in data analyses
RP279	41.331220	-106.502220	COL	Included in data analyses
RP281	40.607780	-108.863000	COL	Included in data analyses
RP285	38.531580	-106.324170	COL	Included in data analyses
RP290	39.007670	-106.998850	COL	Included in data analyses
RP291	39.015050	-107.035200	COL	Included in data analyses
RP294	39.012920	-106.946350	COL	Included in data analyses
RP295	38.998120	-106.981200	COL	Included in data analyses
RP296	38.969920	-107.026730	COL	Included in data analyses
RP301	38.870260	-106.951970	COL	Included in data analyses
RP306	38.866420	-106.912770	COL	Included in data analyses
RP308	38.878800	-106.999120	COL	Included in data analyses
RP482	39.641290	-105.870730	COL	Included in data analyses
RP483	40.198160	-105.541250	COL	Included in data analyses
RP484	39.692450	-105.795840	COL	Included in data analyses
RP485	40.257200	-105.533550	COL	Included in data analyses

RP486	39.353760	-106.217130	COL	Included in data analyses
RP488	39.411700	-106.174610	COL	Included in data analyses
RP489	39.457880	-106.144690	COL	Included in data analyses
RP490	38.767340	-106.319620	COL	Included in data analyses
RP491	40.101220	-105.486010	COL	Included in data analyses
RP492	38.813730	-106.396570	COL	Included in data analyses
RP494	39.762820	-105.729630	COL	Included in data analyses
RP495	39.198920	-106.260550	COL	Included in data analyses
RP496	39.200070	-106.079460	COL	Included in data analyses
RP497	39.237320	-106.293110	COL	Included in data analyses
RP498	39.890790	-105.486350	COL	Included in data analyses
RP499	39.817150	-105.536340	COL	Included in data analyses
RP500	39.612320	-105.716610	COL	Included in data analyses
RP501	39.770820	-105.817910	COL	Included in data analyses
RP502	39.449810	-105.775020	COL	Included in data analyses
RP503	39.067620	-106.505270	COL	Included in data analyses
RP505	39.668870	-105.882540	COL	Included in data analyses
RP506	39.663650	-105.878380	COL	Included in data analyses
RP507	39.660070	-105.871960	COL	Included in data analyses
RP508	39.686850	-105.881510	COL	Included in data analyses
RP510	39.896050	-105.602040	COL	Included in data analyses
RP512	39.866900	-105.595120	COL	Included in data analyses
RP513	39.852180	-105.571110	COL	Included in data analyses
RP514	39.441450	-106.157630	COL	Included in data analyses
RP515	39.604770	-105.909850	COL	Included in data analyses
RP516	40.034590	-105.539950	COL	Included in data analyses
RP522	39.401970	-106.051390	COL	Included in data analyses
RP524	39.908250	-105.556470	COL	Included in data analyses
RP525	39.566590	-105.724130	COL	Included in data analyses
RP527	40.131330	-105.505080	COL	Included in data analyses
RP528	39.362640	-106.311170	COL	Included in data analyses
RP530	39.528680	-106.220160	COL	Included in data analyses
RP532	39.090530	-106.145690	COL	Included in data analyses
RP533	39.471850	-106.043040	COL	Included in data analyses
RP537	37.424100	-108.379220	COL	Included in data analyses
RP538	37.361130	-108.252630	COL	Included in data analyses
RP558	39.835100	-105.411980	COL	Included in data analyses
RP559	39.835100	-105.411980	COL	Included in data analyses
RP560	39.835100	-105.411980	COL	Included in data analyses
RP561	39.835100	-105.411980	COL	Included in data analyses
RP562	39.835100	-105.411980	COL	Included in data analyses
RP563	39.835100	-105.411980	COL	Included in data analyses

RP564	39.835100	-105.411980	COL	Included in data analyses
RP565	39.835100	-105.411980	COL	Included in data analyses
RP566	39.796340	-105.775810	COL	Included in data analyses
RP567	39.796340	-105.775810	COL	Included in data analyses
RP568	39.796340	-105.775810	COL	Included in data analyses
RP569	39.796340	-105.775810	COL	Included in data analyses
RP581	38.361133	-108.252633	COL	Included in data analyses
RP591	38.440633	-107.642283	COL	Included in data analyses
RP592	37.648167	-106.651917	COL	Included in data analyses
RP593	37.646750	-106.588833	COL	Included in data analyses
RP595	37.579183	-108.157050	COL	Included in data analyses
RP596	37.369417	-108.078667	COL	Included in data analyses
RP598	37.600883	-105.212100	COL	Included in data analyses
RP600	37.381033	-108.077367	COL	Included in data analyses
RP601	37.780067	-106.818183	COL	Included in data analyses
RP602	37.796117	-106.801650	COL	Included in data analyses
RP603	37.754867	-108.007050	COL	Included in data analyses
RP604	37.616283	-106.675783	COL	Included in data analyses
RP605	38.332333	-108.107483	COL	Included in data analyses
RP606	38.319467	-108.140000	COL	Included in data analyses
RP607	38.486833	-108.573067	COL	Included in data analyses
RP608	38.521100	-108.603317	COL	Included in data analyses
RP609	38.596617	-108.655700	COL	Included in data analyses
RP610	38.655950	-108.699867	COL	Included in data analyses
RP611	37.485433	-106.768533	COL	Included in data analyses
RP622	39.950023	-105.504519	COL	Included in data analyses
RP623	38.702642	-106.349831	COL	Included in data analyses
RP624	40.017370	-105.532090	COL	Included in data analyses
RP625	38.987883	-106.429597	COL	Included in data analyses
RP626	38.422137	-106.132842	COL	Included in data analyses
RP627	39.274043	-106.309739	COL	Included in data analyses
RP629	39.327870	-106.059082	COL	Included in data analyses
RP630	39.888743	-105.609193	COL	Included in data analyses
RP631	39.904039	-105.497315	COL	Included in data analyses
RP632	39.588520	-106.044552	COL	Included in data analyses
RP633	39.626107	-105.708153	COL	Included in data analyses
RP643	38.285100	-107.367000	COL	Included in data analyses
RP650	39.012517	-108.234950	COL	Included in data analyses
RP651	39.025367	-108.223650	COL	Included in data analyses
RP652	39.036417	-108.187733	COL	Included in data analyses
RP653	39.006050	-108.199717	COL	Included in data analyses
RP654	39.004067	-108.145583	COL	Included in data analyses

RP655	39.004283	-108.110100	COL	Included in data analyses
RP656	39.025817	-108.073600	COL	Included in data analyses
RP657	39.059633	-108.054067	COL	Included in data analyses
RP658	39.049967	-108.081033	COL	Included in data analyses
RP659	39.046100	-108.068117	COL	Included in data analyses
RP660	39.028133	-108.032517	COL	Included in data analyses
RP661	39.030250	-107.996333	COL	Included in data analyses
RP662	39.003550	-107.940917	COL	Included in data analyses
HZ015	44.970670	-114.085850	NOR	Included in data analyses
HZ024	44.964830	-114.074620	NOR	Included in data analyses
HZ037	44.962670	-114.069700	NOR	Included in data analyses
HZ057	44.581880	-114.416730	NOR	Included in data analyses
HZ062	44.581880	-114.416730	NOR	Included in data analyses
HZ068	44.397000	-114.391170	NOR	Included in data analyses
HZ104	44.457280	-114.752880	NOR	Included in data analyses
HZ110	44.457280	-114.752880	NOR	Included in data analyses
HZ117	44.475000	-114.730700	NOR	Included in data analyses
HZ121	44.475000	-114.730700	NOR	Included in data analyses
HZ140	44.494000	-114.734000	NOR	Included in data analyses
HZ143	44.494000	-114.734000	NOR	Included in data analyses
HZ156	44.503000	-114.753200	NOR	Included in data analyses
HZ160	44.457280	-114.752880	NOR	Included in data analyses
HZ170	44.581880	-114.416730	NOR	Included in data analyses
HZ300	44.792383	-113.776183	NOR	Included in data analyses
HZ334	44.797467	-113.803217	NOR	Included in data analyses
HZ337	44.792600	-113.778883	NOR	Included in data analyses
HZ349	45.083733	-114.089667	NOR	Included in data analyses
HZ374	44.861967	-114.295217	NOR	Included in data analyses
HZ444	44.747100	-114.260550	NOR	Included in data analyses
RP003	44.620000	-114.514100	NOR	Included in data analyses
RP010	44.622000	-114.496800	NOR	Included in data analyses
RP014	41.844000	-115.447300	NOR	Included in data analyses
RP016	43.792000	-114.573700	NOR	Included in data analyses
RP024	44.355000	-113.262300	NOR	Included in data analyses
RP035	44.615620	-114.518430	NOR	Included in data analyses
RP048	44.616000	-114.518400	NOR	Included in data analyses
RP049	44.941000	-113.847400	NOR	Included in data analyses
RP054	44.591600	-114.453300	NOR	Included in data analyses
RP059	44.346000	-113.265000	NOR	Included in data analyses
RP061	44.411200	-113.417500	NOR	Included in data analyses
RP064	44.674000	-114.558200	NOR	Included in data analyses
RP069	44.948000	-114.074100	NOR	Included in data analyses

RP078	44.967000	-114.085300	NOR	Included in data analyses
RP079	44.494000	-114.734000	NOR	Included in data analyses
RP080	44.966300	-114.085400	NOR	Included in data analyses
RP082	44.364000	-113.234000	NOR	Included in data analyses
RP087	44.182600	-113.742900	NOR	Included in data analyses
RP088	42.139000	-114.208500	NOR	Included in data analyses
RP091	42.163000	-114.263800	NOR	Included in data analyses
RP094	44.754000	-114.679400	NOR	Included in data analyses
RP099	42.144000	-114.242800	NOR	Included in data analyses
RP101	44.027000	-113.453000	NOR	Included in data analyses
RP103	44.606900	-114.533900	NOR	Included in data analyses
RP104	44.426000	-112.894500	NOR	Included in data analyses
RP107	44.660000	-114.542400	NOR	Included in data analyses
RP108	44.142000	-113.722200	NOR	Included in data analyses
RP116	44.642000	-114.529000	NOR	Included in data analyses
RP117	44.962000	-114.073300	NOR	Included in data analyses
RP118	44.974300	-114.085000	NOR	Included in data analyses
RP121	44.374500	-113.388300	NOR	Included in data analyses
RP127	43.804000	-114.643100	NOR	Included in data analyses
RP132	44.643500	-114.541600	NOR	Included in data analyses
RP135	45.551000	-113.774200	NOR	Included in data analyses
RP138	44.963000	-114.655800	NOR	Included in data analyses
RP140	42.322000	-113.619400	NOR	Included in data analyses
RP141	44.551000	-112.616800	NOR	Included in data analyses
RP159	44.492000	-115.007000	NOR	Included in data analyses
RP160	44.390400	-113.418300	NOR	Included in data analyses
RP162	43.875000	-114.714900	NOR	Included in data analyses
RP166	44.410000	-114.407700	NOR	Included in data analyses
RP169	44.457000	-114.752900	NOR	Included in data analyses
RP173	41.801000	-115.479000	NOR	Included in data analyses
RP179	44.791000	-113.312600	NOR	Included in data analyses
RP184	43.839000	-114.294700	NOR	Included in data analyses
RP185	43.931000	-114.852800	NOR	Included in data analyses
RP187	41.746000	-115.467600	NOR	Included in data analyses
RP197	44.348000	-113.476100	NOR	Included in data analyses
RP203	44.729000	-114.332300	NOR	Included in data analyses
RP204	44.970600	-114.086300	NOR	Included in data analyses
RP206	44.231600	-113.838100	NOR	Included in data analyses
RP213	44.536000	-112.618000	NOR	Included in data analyses
RP216	44.718000	-114.621900	NOR	Included in data analyses
RP235	44.376300	-113.412700	NOR	Included in data analyses
RP237	44.971100	-114.170900	NOR	Included in data analyses

RP239	44.964300	-114.193100	NOR	Included in data analyses
RP240	44.737600	-114.297700	NOR	Included in data analyses
RP241	44.683500	-114.342500	NOR	Included in data analyses
RP242	44.692800	-114.335300	NOR	Included in data analyses
RP248	44.773400	-114.248800	NOR	Included in data analyses
RP251	44.741400	-114.264100	NOR	Included in data analyses
RP256	44.727000	-114.637400	NOR	Included in data analyses
RP258	44.622500	-114.513700	NOR	Included in data analyses
RP259	44.621100	-114.507900	NOR	Included in data analyses
RP260	44.354700	-113.328800	NOR	Included in data analyses
RP264	44.589700	-114.448000	NOR	Included in data analyses
RP266	44.305100	-113.312800	NOR	Included in data analyses
RP311	44.187667	-113.753333	NOR	Included in data analyses
RP312	44.189550	-113.743433	NOR	Included in data analyses
RP313	44.188417	-113.746650	NOR	Included in data analyses
RP314	44.183780	-113.726620	NOR	Included in data analyses
RP316	44.194650	-113.741267	NOR	Included in data analyses
RP317	44.181767	-113.751750	NOR	Included in data analyses
RP319	44.176480	-113.771370	NOR	Included in data analyses
RP320	44.172700	-113.770180	NOR	Included in data analyses
RP321	44.197200	-113.756800	NOR	Included in data analyses
RP322	44.185167	-113.766000	NOR	Included in data analyses
RP323	44.179833	-113.775167	NOR	Included in data analyses
RP476	44.252400	-113.120780	NOR	Included in data analyses
RP477	44.589150	-113.456170	NOR	Included in data analyses
RP616	44.402830	-114.383170	NOR	Included in data analyses
RP617	44.193400	-113.736220	NOR	Included in data analyses
RP621	44.576540	-113.470870	NOR	Included in data analyses
RP644	43.812000	-114.619900	NOR	Included in data analyses
RP008	40.435000	-111.616000	UTA	Included in data analyses
RP012	40.684000	-110.931800	UTA	Included in data analyses
RP075	40.736000	-110.867600	UTA	Included in data analyses
RP093	38.793000	-116.859200	UTA	Included in data analyses
RP095	42.596000	-110.508300	UTA	Included in data analyses
RP143	42.485000	-110.545000	UTA	Included in data analyses
RP145	40.612000	-115.370900	UTA	Included in data analyses
RP165	36.406000	-112.091700	UTA	Included in data analyses
RP181	37.612000	-112.830300	UTA	Included in data analyses
RP190	41.922000	-111.478300	UTA	Included in data analyses
RP196	40.823000	-110.863400	UTA	Included in data analyses
RP207	37.516000	-112.704200	UTA	Included in data analyses
RP214	38.966000	-111.572200	UTA	Included in data analyses

RP219	38.965000	-112.098800	UTA	Included in data analyses
RP236	39.765000	-110.916300	UTA	Included in data analyses
RP280	43.542410	-109.837000	UTA	Included in data analyses
RP324	38.211250	-112.425870	UTA	Included in data analyses
RP325	37.879000	-111.870430	UTA	Included in data analyses
RP326	38.004970	-111.712600	UTA	Included in data analyses
RP327	39.883470	-110.775620	UTA	Included in data analyses
RP328	39.879030	-110.799700	UTA	Included in data analyses
RP329	39.880280	-110.760320	UTA	Included in data analyses
RP330	39.872700	-110.817670	UTA	Included in data analyses
RP331	40.313620	-111.280250	UTA	Included in data analyses
RP332	38.652450	-111.946350	UTA	Included in data analyses
RP333	38.096530	-111.692250	UTA	Included in data analyses
RP335	38.475130	-111.460830	UTA	Included in data analyses
RP337	37.945220	-112.201850	UTA	Included in data analyses
RP338	40.218930	-111.250430	UTA	Included in data analyses
RP339	37.984750	-112.223980	UTA	Included in data analyses
RP340	38.630220	-111.979830	UTA	Included in data analyses
RP342	37.944030	-111.699530	UTA	Included in data analyses
RP344	37.980120	-111.724720	UTA	Included in data analyses
RP345	38.070080	-112.141700	UTA	Included in data analyses
RP346	39.084830	-111.339730	UTA	Included in data analyses
RP347	39.080580	-111.318780	UTA	Included in data analyses
RP348	38.509300	-111.496680	UTA	Included in data analyses
RP349	38.814330	-111.667250	UTA	Included in data analyses
RP350	38.806530	-111.689930	UTA	Included in data analyses
RP352	37.928720	-111.866180	UTA	Included in data analyses
RP353	37.830530	-111.882770	UTA	Included in data analyses
RP354	40.537820	-110.355750	UTA	Included in data analyses
RP355	38.448200	-111.449330	UTA	Included in data analyses
RP356	38.626880	-111.647320	UTA	Included in data analyses
RP357	38.675020	-111.943020	UTA	Included in data analyses
RP358	37.959850	-111.838320	UTA	Included in data analyses
RP359	38.059770	-111.591300	UTA	Included in data analyses
RP360	38.532480	-111.952630	UTA	Included in data analyses
RP361	38.562730	-111.967580	UTA	Included in data analyses
RP362	38.550420	-111.971520	UTA	Included in data analyses
RP363	38.577000	-111.945950	UTA	Included in data analyses
RP364	38.591430	-111.939600	UTA	Included in data analyses
RP365	38.662600	-112.275270	UTA	Included in data analyses
RP366	40.294120	-111.251300	UTA	Included in data analyses
RP367	38.496280	-111.458130	UTA	Included in data analyses

RP368	40.564980	-110.504520	UTA	Included in data analyses
RP369	40.554980	-110.488730	UTA	Included in data analyses
RP370	40.533780	-110.464380	UTA	Included in data analyses
RP372	38.752680	-111.651880	UTA	Included in data analyses
RP373	38.723950	-111.678070	UTA	Included in data analyses
RP374	39.812600	-111.694570	UTA	Included in data analyses
RP375	39.834480	-111.722150	UTA	Included in data analyses
RP376	39.842420	-111.721770	UTA	Included in data analyses
RP377	39.879080	-111.685620	UTA	Included in data analyses
RP378	39.919080	-111.633620	UTA	Included in data analyses
RP379	40.028280	-111.258330	UTA	Included in data analyses
RP380	38.696380	-111.937900	UTA	Included in data analyses
RP381	39.314100	-111.366730	UTA	Included in data analyses
RP382	38.679600	-112.285850	UTA	Included in data analyses
RP383	38.220570	-112.414350	UTA	Included in data analyses
RP384	38.066270	-111.633380	UTA	Included in data analyses
RP386	39.332150	-111.433100	UTA	Included in data analyses
RP387	39.319970	-111.410320	UTA	Included in data analyses
RP388	39.385000	-111.387550	UTA	Included in data analyses
RP389	39.305380	-111.389820	UTA	Included in data analyses
RP390	39.319180	-111.473830	UTA	Included in data analyses
RP391	39.906880	-111.241650	UTA	Included in data analyses
RP392	39.883330	-111.263830	UTA	Included in data analyses
RP393	39.864450	-111.286530	UTA	Included in data analyses
RP396	39.748900	-111.323550	UTA	Included in data analyses
RP398	39.986580	-111.153970	UTA	Included in data analyses
RP399	40.036370	-111.200200	UTA	Included in data analyses
RP400	40.585650	-110.330550	UTA	Included in data analyses
RP401	40.224070	-111.228830	UTA	Included in data analyses
RP402	38.609980	-111.671650	UTA	Included in data analyses
RP404	38.581400	-111.478930	UTA	Included in data analyses
RP405	38.254550	-112.331970	UTA	Included in data analyses
RP407	38.263850	-112.380530	UTA	Included in data analyses
RP408	38.225680	-112.439420	UTA	Included in data analyses
RP409	38.675050	-111.662680	UTA	Included in data analyses
RP410	40.919820	-110.767530	UTA	Included in data analyses
RP411	40.935570	-110.704130	UTA	Included in data analyses
RP413	40.950600	-110.647350	UTA	Included in data analyses
RP414	40.956930	-110.675970	UTA	Included in data analyses
RP415	40.929350	-110.614980	UTA	Included in data analyses
RP417	40.896700	-110.662030	UTA	Included in data analyses
RP418	40.947070	-110.582900	UTA	Included in data analyses

RP419	40.932730	-110.562520	UTA	Included in data analyses
RP420	40.909980	-110.548250	UTA	Included in data analyses
RP421	40.956700	-110.558850	UTA	Included in data analyses
RP422	40.957070	-110.503900	UTA	Included in data analyses
RP423	40.953030	-110.461350	UTA	Included in data analyses
RP424	40.942930	-110.417500	UTA	Included in data analyses
RP425	40.923120	-110.404980	UTA	Included in data analyses
RP426	40.954520	-110.392980	UTA	Included in data analyses
RP427	41.000830	-110.378100	UTA	Included in data analyses
RP428	41.010830	-110.356950	UTA	Included in data analyses
RP429	40.981900	-110.345420	UTA	Included in data analyses
RP430	40.954280	-110.317370	UTA	Included in data analyses
RP432	37.972720	-111.816700	UTA	Included in data analyses
RP433	38.612550	-111.967950	UTA	Included in data analyses
RP434	38.051880	-112.168050	UTA	Included in data analyses
RP435	40.602000	-110.347020	UTA	Included in data analyses
RP453	44.992280	-111.858130	UTA	Included in data analyses
RP469	41.452220	-111.504370	UTA	Included in data analyses
RP470	41.417750	-111.521330	UTA	Included in data analyses
RP471	41.475320	-111.483930	UTA	Included in data analyses
RP635	40.929350	-110.614980	UTA	Included in data analyses
RP638	37.975267	-112.217267	UTA	Included in data analyses
RP645	39.825100	-111.356450	UTA	Included in data analyses
RP646	39.693330	-111.328280	UTA	Included in data analyses
RP647	38.455650	-111.452630	UTA	Included in data analyses
RP648	38.223500	-112.376600	UTA	Included in data analyses
RP668	38.014300	-111.761620	UTA	Included in data analyses
RP669	38.478780	-111.459550	UTA	Included in data analyses
RP670	37.999050	-111.794770	UTA	Included in data analyses
HZ041	45.033480	-114.106450	WES	Included in data analyses
HZ053	45.126580	-114.136630	WES	Included in data analyses
HZ088	44.443380	-114.734130	WES	Included in data analyses
HZ097	44.443380	-114.734130	WES	Included in data analyses
HZ102	44.457280	-114.752880	WES	Included in data analyses
HZ152	44.503000	-114.753200	WES	Included in data analyses
HZ365	45.114550	-114.078250	WES	Included in data analyses
HZ411	44.794300	-114.258367	WES	Included in data analyses
HZ422	44.832783	-114.254700	WES	Included in data analyses
HZ449	44.441583	-114.733583	WES	Included in data analyses
RP017	45.654400	-113.786700	WES	Included in data analyses
RP021	37.913000	-119.257600	WES	Included in data analyses
RP033	43.497000	-110.954400	WES	Included in data analyses

RP037	44.406000	-115.285800	WES	Included in data analyses
RP041	44.965000	-114.602100	WES	Included in data analyses
RP046	44.366000	-115.143500	WES	Included in data analyses
RP053	44.288000	-115.481300	WES	Included in data analyses
RP070	45.710000	-113.998100	WES	Included in data analyses
RP072	45.705000	-113.988700	WES	Included in data analyses
RP074	45.703000	-113.985200	WES	Included in data analyses
RP076	44.941000	-114.121100	WES	Included in data analyses
RP089	45.200000	-115.990200	WES	Included in data analyses
RP092	44.912000	-114.386600	WES	Included in data analyses
RP098	44.444000	-115.089300	WES	Included in data analyses
RP105	44.604000	-114.542400	WES	Included in data analyses
RP106	45.720000	-113.994200	WES	Included in data analyses
RP109	45.547000	-113.763200	WES	Included in data analyses
RP114	45.336000	-114.003900	WES	Included in data analyses
RP115	45.141000	-113.469500	WES	Included in data analyses
RP120	45.125000	-114.147200	WES	Included in data analyses
RP122	44.841300	-114.272300	WES	Included in data analyses
RP126	44.865000	-114.298000	WES	Included in data analyses
RP128	45.115500	-114.144200	WES	Included in data analyses
RP130	45.534000	-113.080000	WES	Included in data analyses
RP131	38.051000	-119.250200	WES	Included in data analyses
RP137	44.968000	-114.121000	WES	Included in data analyses
RP147	44.475000	-114.730700	WES	Included in data analyses
RP148	48.706000	-120.672200	WES	Included in data analyses
RP149	43.768000	-113.901400	WES	Included in data analyses
RP150	45.679000	-113.832800	WES	Included in data analyses
RP154	36.449000	-118.228900	WES	Included in data analyses
RP161	46.268000	-111.927500	WES	Included in data analyses
RP167	44.416000	-115.470300	WES	Included in data analyses
RP174	45.156400	-114.151700	WES	Included in data analyses
RP183	44.855000	-118.263800	WES	Included in data analyses
RP188	44.807000	-114.270600	WES	Included in data analyses
RP192	44.411000	-115.372000	WES	Included in data analyses
RP193	44.795000	-113.782200	WES	Included in data analyses
RP200	44.296000	-115.479900	WES	Included in data analyses
RP202	45.118500	-114.158400	WES	Included in data analyses
RP210	44.834000	-118.247700	WES	Included in data analyses
RP223	44.370000	-115.397000	WES	Included in data analyses
RP224	45.442500	-113.838400	WES	Included in data analyses
RP225	45.095000	-113.423300	WES	Included in data analyses
RP227	44.479000	-115.320400	WES	Included in data analyses

RP230	44.503000	-114.753200	WES	Included in data analyses
RP232	44.702500	-114.315800	WES	Included in data analyses
RP233	45.602200	-113.682500	WES	Included in data analyses
RP244	46.561000	-112.303200	WES	Included in data analyses
RP249	44.840900	-114.274200	WES	Included in data analyses
RP254	45.170000	-113.520500	WES	Included in data analyses
RP263	45.569700	-113.706500	WES	Included in data analyses
RP439	45.347120	-111.529670	WES	Included in data analyses
RP445	45.458430	-111.854370	WES	Included in data analyses
RP448	44.923220	-111.359380	WES	Included in data analyses
RP450	45.125250	-111.862000	WES	Included in data analyses
RP461	44.562700	-112.143000	WES	Included in data analyses
RP462	44.541780	-112.126520	WES	Included in data analyses
RP464	44.596780	-111.536120	WES	Included in data analyses
RP468	44.485270	-112.088370	WES	Included in data analyses
RP472	37.434480	-118.747720	WES	Included in data analyses
RP473	37.431330	-118.749470	WES	Included in data analyses
RP474	37.433970	-118.748000	WES	Included in data analyses
RP475	37.743950	-119.035770	WES	Included in data analyses
RP479	44.643680	-113.531830	WES	Included in data analyses
RP480	44.608120	-113.576270	WES	Included in data analyses
RP636	44.843450	-114.272933	WES	Included in data analyses
RP641	44.886700	-111.356280	WES	Included in data analyses
RP649	44.577030	-113.472080	WES	Included in data analyses
XX044	38.838818	-106.413099	NA	excluded from data analyes
RP597	37.394750	-108.068800	NA	excluded from data analyes
XX063	37.984970	-111.719630	NA	excluded from data analyes
RP180	unknown	unknown	NA	excluded from data analyes
XX004	38.813730	-106.396570	NA	excluded from data analyes
RP182	unknown	unknown	NA	excluded from data analyes
RP467	44.411030	-112.001280	NA	excluded from data analyes
RP198	44.421000	-112.876000	NA	excluded from data analyes
RP018	44.453000	-112.916300	NA	excluded from data analyes
RP442	44.464200	-111.998530	NA	excluded from data analyes
RP466	44.490630	-111.654570	NA	excluded from data analyes
RP441	44.493550	-111.767300	NA	excluded from data analyes
RP084	44.523000	-112.192500	NA	excluded from data analyes
RP215	44.556000	-112.639000	NA	excluded from data analyes
RP463	44.569920	-112.020600	NA	excluded from data analyes
RP465	44.601970	-111.496800	NA	excluded from data analyes
RP444	44.675770	-111.276980	NA	excluded from data analyes
RP459	44.862220	-111.621930	NA	excluded from data analyes

RP457	44.884030	-111.752620	NA	excluded from data analyes
RP456	44.898120	-111.780120	NA	excluded from data analyes
XX064	44.956270	-111.859980	NA	excluded from data analyes
XX059	45.072133	-114.085823	NA	excluded from data analyes
RP449	45.141950	-111.834970	NA	excluded from data analyes
RP209	45.537000	-113.829100	NA	excluded from data analyes
RP211	45.541000	-113.826500	NA	excluded from data analyes
RP218	45.634000	-112.925000	NA	excluded from data analyes
RP097	45.853000	-113.438500	NA	excluded from data analyes
RP158	45.870000	-113.226400	NA	excluded from data analyes
RP270b	44.300750	-107.305780	NA	excluded from data analyes
RP670b	37.999050	-111.794770	NA	excluded from data analyes
RP669b	38.478780	-111.459550	NA	excluded from data analyes
RP668b	38.014300	-111.761620	NA	excluded from data analyes
RP365b	38.662600	-112.275270	NA	excluded from data analyes

NA, not applicable

Table S2 Genotype accuracy estimated by comparing called SNPs to Sanger sequences

SampleID	Alias*	Latitude	Longitude	Mean depth	Identical genotypes	Different genotypes	False%
RP072	ID06_04	45.705000	-113.988700	2.7544	413	0	0
RP148	WA26_17	48.706000	-120.672200	3.1167	814	2	0.245098
RP094	ID73	44.754000	-114.679400	3.0629	829	1	0.120482
RP184	ID87_44B	43.839000	-114.294700	3.0746	832	0	0
RP109	MT55	45.547000	-113.763200	3.0754	868	2	0.229885
RP010	ID14_40B	44.622000	-114.496800	3.1079	875	1	0.114155
RP165	AZ01_06	36.406000	-112.091700	3.0769	918	5	0.541712
RP149	ID116_3A	43.768000	-113.901400	3.0875	942	3	0.31746
RP042	CO22_01	40.001000	-105.468100	3.271	942	0	0
RP179	ID120_1B	44.791000	-113.312600	3.2684	956	1	0.104493
RP073	CO21_12	39.951000	-105.593600	3.1889	1018	2	0.196078
RP181	UT05_09	37.612000	-112.830300	3.2535	1042	1	0.095877
RP032	CO27_05	40.399630	-106.119000	3.3152	1083	0	0
RP139	CO29_01	40.544000	-106.625000	3.4399	1122	1	0.089047
RP213	MT51	44.536000	-112.618000	3.4841	1176	1	0.084962
RP067	SAD12	38.707000	-106.804500	3.6365	1202	0	0
RP125	CO16_03	39.684000	-105.607100	3.4356	1238	0	0
RP154	CA24_11	36.449000	-118.228900	4.4723	1331	0	0
RP197	ID86_25A	44.348000	-113.476100	3.786	1351	1	0.073964
RP152	CO18_4	39.864000	-105.444700	3.8295	1363	1	0.073314
RP195	UT07_05	37.807600	-109.818700	5.869	1765	2	0.113186
Average							0.114272

*Sample name in Song et al. (2009)

Length of sanger sequences is 69559 bp; Number of SNPs is 1909.

Table S3 Comparison of summary statistics (mean ± standard error) between F_{ST}-islands and the rest of the genome for all group-pairs. *P*-values were estimated by randomization test with 100,000 permutations.

Parameter[a]	Genomic Islands	Genomic background	*P*-value
**COL vs. NOR (*N* = 306)**			
d_{XY}	2.731 ± 0.127	1.583 ± 0.013	$< 1 \times 10^{-5}$
Fay & Wu's *H* (COL)	-0.209 ± 0.013	-0.15 ± 0.003	$< 1 \times 10^{-5}$
Fay & Wu's *H* (NOR)	-0.961 ± 0.033	-0.628 ± 0.007	$< 1 \times 10^{-5}$
F_{ST}	0.559 ± 0.005	0.206 ± 0.001	NA
Gene density	4431.905 ± 203.435	4540.454 ± 35.251	0.5277
Recombination rate (COL)	0.545 ± 0.031	0.636 ± 0.007	0.0076
Recombination rate (NOR)	0.291 ± 0.046	0.254 ± 0.006	0.2128
RND	0.191 ± 0.009	0.116 ± 0.001	$< 1 \times 10^{-5}$
Scaled π (COL)	0.124 ± 0.005	0.14 ± 0.001	0.0046
Scaled π (NOR)	0.064 ± 0.004	0.08 ± 0.001	9.00E-04
Tajima's *D* (COL)	-1.062 ± 0.044	-0.941 ± 0.008	0.0025
Tajima's *D* (NOR)	-0.949 ± 0.056	-0.393 ± 0.014	$< 1 \times 10^{-5}$
π (COL)	1.682 ± 0.066	1.893 ± 0.016	0.0055
π (NOR)	0.901 ± 0.055	1.084 ± 0.013	0.0026
**COL vs. UTA (*N* = 349)**			
d_{XY}	2.278 ± 0.086	1.8 ± 0.015	$< 1 \times 10^{-5}$
Fay & Wu's *H* (COL)	-0.138 ± 0.01	-0.15 ± 0.003	0.3284
Fay & Wu's *H* (UTA)	-0.37 ± 0.017	-0.273 ± 0.004	$< 1 \times 10^{-5}$
F_{ST}	0.385 ± 0.004	0.124 ± 0.001	NA
Gene density	4611 ± 154.357	4450.138 ± 35.599	0.3141
Recombination rate (COL)	0.624 ± 0.036	0.614 ± 0.007	0.7536
Recombination rate (UTA)	0.718 ± 0.043	0.901 ± 0.013	0.0026
RND	0.159 ± 0.006	0.126 ± 0.001	$< 1 \times 10^{-5}$
Scaled π (COL)	0.127 ± 0.004	0.142 ± 0.001	0.02
Scaled π (UTA)	0.11 ± 0.005	0.138 ± 0.001	2×10^{-4}
Tajima's *D* (COL)	-0.934 ± 0.037	-0.942 ± 0.008	0.8425
Tajima's *D* (UTA)	-1.349 ± 0.047	-1.138 ± 0.009	$< 1 \times 10^{-5}$
π (COL)	1.692 ± 0.059	1.924 ± 0.017	0.0017
π (UTA)	1.52 ± 0.074	1.925 ± 0.02	$< 1 \times 10^{-5}$
**UTA vs. NOR (*N* = 352)**			
d_{XY}	2.522 ± 0.119	1.464 ± 0.014	$< 1 \times 10^{-5}$
Fay & Wu's *H* (UTA)	-0.315 ± 0.017	-0.274 ± 0.004	0.0179
Fay & Wu's *H* (NOR)	-0.896 ± 0.028	-0.629 ± 0.007	$< 1 \times 10^{-5}$
F_{ST}	0.483 ± 0.004	0.152 ± 0.001	NA
Gene density	4160.366 ± 148.297	4551.862 ± 35.847	0.0151
Recombination rate (UTA)	0.735 ± 0.045	0.891 ± 0.013	0.0078
Recombination rate (NOR)	0.191 ± 0.018	0.247 ± 0.006	0.0293
RND	0.165 ± 0.007	0.105 ± 0.001	$< 1 \times 10^{-5}$
Scaled π (UTA)	0.122 ± 0.005	0.135 ± 0.001	0.0228
Scaled π (NOR)	0.066 ± 0.004	0.08 ± 0.001	8×10^{-4}
Tajima's *D* (UTA)	-1.122 ± 0.046	-1.158 ± 0.01	0.393
Tajima's *D* (NOR)	-0.714 ± 0.056	-0.402 ± 0.014	$< 1 \times 10^{-5}$
π (UTA)	1.821 ± 0.084	1.873 ± 0.019	0.5347
π (NOR)	0.933 ± 0.051	1.086 ± 0.013	0.0067
**WES vs. COL (*N* = 163)**			
d_{XY}	4.225 ± 0.187	2.45 ± 0.016	$< 1 \times 10^{-5}$
Fay & Wu's *H* (WES)	-1.243 ± 0.041	-0.895 ± 0.006	$< 1 \times 10^{-5}$
Fay & Wu's *H* (COL)	-0.288 ± 0.024	-0.149 ± 0.003	$< 1 \times 10^{-5}$

F_{ST}	0.777 ± 0.003	0.429 ± 0.002	NA
Gene density	4620.374 ± 316.101	4481.933 ± 34.787	0.544
Recombination rate (WES)	0.422 ± 0.036	0.549 ± 0.009	0.0293
Recombination rate (COL)	0.668 ± 0.072	1.037 ± 0.012	< 1 x 10^{-5}
RND	0.307 ± 0.014	0.176 ± 0.001	< 1 x 10^{-5}
Scaled π (WES)	0.092 ± 0.006	0.098 ± 0.001	0.3426
Scaled π (COL)	0.083 ± 0.004	0.141 ± 0.001	< 1 x 10^{-5}
Tajima's *D* (WES)	-1.119 ± 0.042	-0.783 ± 0.01	< 1 x 10^{-5}
Tajima's *D* (COL)	-1.567 ± 0.049	-0.928 ± 0.008	< 1 x 10^{-5}
π (WES)	1.162 ± 0.056	1.315 ± 0.013	0.0772
π (COL)	1.083 ± 0.053	1.921 ± 0.016	< 1 x 10^{-5}
**WES vs. NOR (*N* = 22)**			
d_{XY}	5.011 ± 0.578	2.27 ± 0.016	< 1 x 10^{-5}
Fay & Wu's *H* (WES)	-1.629 ± 0.112	-0.898 ± 0.006	< 1 x 10^{-5}
Fay & Wu's *H* (NOR)	-1.441 ± 0.176	-0.628 ± 0.007	< 1 x 10^{-5}
F_{ST}	0.901 ± 0.004	0.555 ± 0.002	NA
Gene density	3934.045 ± 618.685	4543.327 ± 34.975	0.3144
Recombination rate (WES)	0.379 ± 0.06	0.554 ± 0.009	0.2464
Recombination rate (NOR)	0.123 ± 0.04	0.418 ± 0.009	0.0631
RND	0.391 ± 0.059	0.164 ± 0.001	< 1 x 10^{-5}
Scaled π (WES)	0.07 ± 0.007	0.097 ± 0.001	0.1002
Scaled π (NOR)	0.03 ± 0.007	0.08 ± 0.001	0.0061
Tajima's *D* (WES)	-1.48 ± 0.053	-0.789 ± 0.01	5 x 10^{-4}
Tajima's *D* (NOR)	-1.926 ± 0.125	-0.413 ± 0.013	< 1 x 10^{-5}
π (WES)	0.94 ± 0.094	1.298 ± 0.013	0.1062
π (NOR)	0.322 ± 0.032	1.073 ± 0.012	0.0049
**WES vs. UTA (*N* = 85)**			
d_{XY}	5.423 ± 0.363	2.728 ± 0.019	< 1 x 10^{-5}
Fay & Wu's *H* (WES)	-1.319 ± 0.053	-0.895 ± 0.006	< 1 x 10^{-5}
Fay & Wu's *H* (UTA)	-0.576 ± 0.049	-0.273 ± 0.004	< 1 x 10^{-5}
F_{ST}	0.843 ± 0.003	0.489 ± 0.002	NA
Gene density	4621.494 ± 309.24	4476.191 ± 34.985	0.639
Recombination rate (WES)	0.43 ± 0.059	0.549 ± 0.009	0.1457
Recombination rate (UTA)	1.074 ± 0.19	1.487 ± 0.022	0.0438
RND	0.364 ± 0.022	0.19 ± 0.001	< 1 x 10^{-5}
Scaled π (WES)	0.094 ± 0.008	0.098 ± 0.001	0.633
Scaled π (UTA)	0.059 ± 0.005	0.138 ± 0.001	< 1 x 10^{-5}
Tajima's *D* (WES)	-1.034 ± 0.055	-0.788 ± 0.01	0.0075
Tajima's *D* (UTA)	-2.026 ± 0.05	-1.139 ± 0.009	< 1 x 10^{-5}
π (WES)	1.268 ± 0.094	1.313 ± 0.013	0.7003
π (UTA)	0.826 ± 0.063	1.909 ± 0.019	< 1 x 10^{-5}

[a] d_{XY} and π are in unit of 10^{-3}, recombination rate (ρ/π) is scaled by diversity , and gene density is total length of coding regions (bp) per 20-kb window.
N, number of outlier windows.
NA, not test for the extreme of F_{ST} distribution (F_{ST}-islands) to the rest of the distribution.

Table S4 Patterns of isolation by distance in *B. stricta* groups.

		Mantel test		Ratio of increased diversity per 100 km (%)	
Group/species	No. of genotypes	*r*	*P*	Mean	Standard error
NOR	105	0.33	0.0001	18.58	3.53
UTA	126	0.23	0.0001	10.42	2.23
COL	157	0.19	0.0023	3.36	2.69
WES	69	0.36	0.0003	11.95	8.34

Table S5 Relative likelihood of the different models shown in Fig. S3

Model	No. of parameters	$\log_{10}$(Likelihood)[a]	AIC_i[b]	Δ_i[b]	Model normalized relative likelihood (W_i[b])
Model 1	28	-19801333.66	39602723.32	223700.48	0
Model 2	10	-20104883.06	40209786.12	830763.282	0
Model 3	32	-19737204.31	39474472.61	95449.772	0
Model 4	14	-20058425.26	40116878.52	737855.68	0
Model 5	33	-19694093.3	39388252.59	9229.754	0
Model 6	15	-20049950.39	40099930.78	720907.942	0
Model 7	31	-19707142.23	39414346.46	35323.616	0
Model 8	13	-20057749.75	40115525.51	736502.668	0
Model 9	34	-19693885.26	39387838.53	8815.686	0
Model 10	16	-20052991.34	40106014.68	726991.838	0
Model 11	38	-19689473.42	39379022.84	0	1
Model 12	20	-20048580.62	40097201.24	718178.398	0
Model 13	38	-19689729.41	39379534.82	511.976	6.6961E-112
Model 14	20	-20047807.53	40095655.06	716632.22	0

[a]The highest likelihood value among the 50 independent runs for each model.

[b]The calculation of AIC_i, Δ_i and w_i are according to the methods shown in Excoffier et al. (2013).

Table S6 Maximum likelihood estimates and 95% confidence interval of parameters in the best-fit model (Model 11) inferred by fastsimcoal2.

Parameters	Point estimate	2.5% Percentile	97.5% Percentile
N_{WES}	795.99	793.51	1086.57
N_{COL}	1112.16	990.07	1277.61
N_{UTA}	500.47	461.11	1466.66
N_{NOR}	132.38	111.14	158.65
N_{WES_bot}	203.14	202.38	560.71
N_{COL_bot}	270.22	237.32	404.77
N_{UTA_bot}	487.66	270.39	644.13
N_{NOR_bot}	13.62	10.28	21.77
N_{WES_sta}	18.64	17.89	19.75
N_{COL_sta}	100.49	99.95	105.26
N_{UTA_sta}	107.57	104.37	132.77
N_{NOR_sta}	11.46	10.46	12.91
N_{ANC}	24.20	22.74	31.50
N_{ANC_CUN}	35.82	33.67	41.37
N_{ANC_UN}	38.83	29.70	44.23
T_S	12.08	11.28	12.31
T_{EXP}	7.69	4.98	9.68
T_{DIV0}	29.99	28.28	30.98
T_{DIV1}	68.54	65.02	72.92
T_{DIV2}	179.33	154.64	185.41
$M_{WES->COL}$	3.20E-06	2.37E-06	3.75E-06
$M_{WES->UTA}$	4.06E-06	2.55E-06	5.75E-06
$M_{WES->NOR}$	2.94E-06	2.01E-06	3.82E-06
$M_{COL->WES}$	4.80E-07	4.37E-07	5.83E-07
$M_{COL->UTA}$	1.01E-05	9.27E-06	1.06E-05
$M_{COL->NOR}$	8.79E-07	3.85E-07	9.83E-07
$M_{UTA->WES}$	3.76E-07	2.69E-07	4.59E-07
$M_{UTA->COL}$	1.03E-05	9.53E-06	1.09E-05
$M_{UTA->NOR}$	4.11E-06	3.38E-06	4.64E-06
$M_{NOR->WES}$	4.64E-06	4.32E-06	5.17E-06
$M_{NOR->COL}$	8.94E-06	7.54E-06	9.58E-06
$M_{NOR->UTA}$	1.95E-05	1.74E-05	2.12E-05
$M_{WES->UN}$	6.84E-10	5.78E-10	9.20E-10
$M_{UN->WES}$	1.65E-07	1.35E-07	8.49E-07
$M_{COL->UN}$	3.19E-07	1.57E-07	8.99E-07
$M_{UN->COL}$	1.75E-05	8.82E-06	3.08E-05
$M_{WES->CUN}$	7.09E-09	1.92E-09	9.86E-09
$M_{CUN->WES}$	6.91E-06	4.43E-06	7.56E-06

Parameter tags corresponding to the model shown in Fig. S4.

Parameters with N denote effective population sizes of current and ancestral populations, with T time estimates of divergence (T_{DIV0}, T_{DIV1} and T_{DIV2}) and population expenexpansion (T_{EXP}), with M migration between groups.

T_S is time of instantaneous size change.

Estimates of effective population size and time are scaled by 10^3 individuals and thousand year ago (KYA), respectively.

Gene flow estimated between groups is given in migration rate per generation.

Table S7 Nucleotide diversity (π, mean ± standard error, in unit of 10^{-3}) in different genomic regions, and number and size of genomic islands in each group.

Group	Genomic background	All islands	Shared islands	No. of 20-kb outliers
COL	1.807 ± 0.012	7.84 ± 0.123	8.939 ± 0.211	310
NOR	1.014 ± 0.009	6.037 ± 0.111	7.195 ± 0.183	315
UTA	1.791 ± 0.014	9.025 ± 0.142	10.126 ± 0.233	307
WES	1.240 ± 0.009	6.442 ± 0.103	7.553 ± 0.186	345

Table S8 Number/ratio of 20-kb high diversity outlier windows and NBS-LRR genes in three genomic regions.

	Outliers in WES	Outlier in COL	Outlier in UTA	Outlier in NOR	Shared outliers	LRR-NBS
LG1p	101/0.293	81/0.261	84/0.274	92/0.292	53/0.41	132/0.35
LG5p	28/0.081	27/0.087	26/0.085	31/0.098	17/0.13	49/0.13
LG7p	54/0.157	35/0.113	39/0.127	33/0.105	21/0.16	46/0.12
Total	183/0.53	143/0.461	149/0.485	156/0.495	91/0.70	227/0.60

NOTE, ratio = number of outlier windows or LRR-NBS genes in each genomic region divided by total number of outliers or LRR-NBS genes.

Table S9 Comparison of summary statistics (mean ± standard error) between the three genomic regions and the genomic background. *P*-values were estimated by randomization test with 100,000 permutations.

Parameter[a]	With outliers	Without outliers	Background	*P*-value (with outliers)	*P*-value (without outliers)
**LG1p**					
π (COL)	4.789 ± 0.168	3.183 ± 0.086	1.743 ± 0.011	$< 1 \times 10^{-5}$	$< 1 \times 10^{-5}$
π (NOR)	3.743 ± 0.161	2.119 ± 0.066	0.965 ± 0.008	$< 1 \times 10^{-5}$	$< 1 \times 10^{-5}$
π (UTA)	5.415 ± 0.205	3.438 ± 0.109	1.707 ± 0.013	$< 1 \times 10^{-5}$	$< 1 \times 10^{-5}$
π (WES)	4.204 ± 0.167	2.425 ± 0.077	1.185 ± 0.009	$< 1 \times 10^{-5}$	$< 1 \times 10^{-5}$
Scaled π (COL)	0.37 ± 0.019	0.236 ± 0.011	0.131 ± 0.001	$< 1 \times 10^{-5}$	$< 1 \times 10^{-5}$
Scaled π (NOR)	0.301 ± 0.019	0.152 ± 0.007	0.073 ± 0.001	$< 1 \times 10^{-5}$	$< 1 \times 10^{-5}$
Scaled π (UTA)	0.404 ± 0.021	0.248 ± 0.012	0.126 ± 0.001	$< 1 \times 10^{-5}$	$< 1 \times 10^{-5}$
Scaled π (WES)	0.32 ± 0.016	0.169 ± 0.007	0.09 ± 0.001	$< 1 \times 10^{-5}$	$< 1 \times 10^{-5}$
Tajima's *D* (COL)	-0.469 ± 0.042	-0.666 ± 0.044	-0.987 ± 0.007	$< 1 \times 10^{-5}$	$< 1 \times 10^{-5}$
Tajima's *D* (NOR)	0.153 ± 0.06	-0.083 ± 0.07	-0.456 ± 0.013	$< 1 \times 10^{-5}$	$< 1 \times 10^{-5}$
Tajima's *D* (UTA)	-0.631 ± 0.044	-0.832 ± 0.046	-1.2 ± 0.009	$< 1 \times 10^{-5}$	$< 1 \times 10^{-5}$
Tajima's *D* (WES)	-0.13 ± 0.05	-0.41 ± 0.054	-0.835 ± 0.009	$< 1 \times 10^{-5}$	$< 1 \times 10^{-5}$
Fay & Wu's *H* (COL)	-0.148 ± 0.016	-0.178 ± 0.02	-0.15 ± 0.003	0.8878	0.0905
Fay & Wu's *H* (NOR)	-0.39 ± 0.033	-0.521 ± 0.041	-0.635 ± 0.007	$< 1 \times 10^{-5}$	0.0103
Fay & Wu's *H* (UTA)	-0.268 ± 0.025	-0.359 ± 0.032	-0.276 ± 0.004	0.6731	8×10^{-4}
Fay & Wu's *H* (WES)	-0.445 ± 0.031	-0.562 ± 0.033	-0.912 ± 0.006	$< 1 \times 10^{-5}$	$< 1 \times 10^{-5}$
Gene density	3928.015 ± 163.988	3687.723 ± 178.187	4560.753 ± 35.436	5×10^{-4}	1×10^{-4}
d_{XY} (COL vs. NOR)	4.025 ± 0.139	2.474 ± 0.067	1.472 ± 0.009	$< 1 \times 10^{-5}$	$< 1 \times 10^{-5}$
d_{XY} (COL vs. UTA)	4.381 ± 0.151	2.661 ± 0.079	1.622 ± 0.01	$< 1 \times 10^{-5}$	$< 1 \times 10^{-5}$
$\mathrm{d_{XY}}$ (UTA vs. NOR)	4.227 ± 0.155	2.479 ± 0.075	1.338 ± 0.009	$< 1 \times 10^{-5}$	$< 1 \times 10^{-5}$
d_{XY} (WES vs. COL)	4.858 ± 0.156	3.213 ± 0.097	2.31 ± 0.013	$< 1 \times 10^{-5}$	$< 1 \times 10^{-5}$
d_{XY} (WES vs. NOR)	4.416 ± 0.143	2.846 ± 0.086	2.125 ± 0.012	$< 1 \times 10^{-5}$	$< 1 \times 10^{-5}$
d_{XY} (WES vs. UTA)	5.393 ± 0.169	3.512 ± 0.103	2.537 ± 0.014	$< 1 \times 10^{-5}$	$< 1 \times 10^{-5}$
RND (COL vs. NOR)	0.301 ± 0.015	0.161 ± 0.007	0.106 ± 0.001	$< 1 \times 10^{-5}$	$< 1 \times 10^{-5}$
RND (COL vs. UTA)	0.316 ± 0.015	0.168 ± 0.007	0.114 ± 0.001	$< 1 \times 10^{-5}$	$< 1 \times 10^{-5}$
RND (UTA vs. NOR)	0.311 ± 0.016	0.16 ± 0.007	0.094 ± 0.001	$< 1 \times 10^{-5}$	$< 1 \times 10^{-5}$
RND (WES vs. COL)	0.356 ± 0.015	0.208 ± 0.009	0.166 ± 0.001	$< 1 \times 10^{-5}$	$< 1 \times 10^{-5}$
RND (WES vs. NOR)	0.328 ± 0.015	0.187 ± 0.008	0.155 ± 0.001	$< 1 \times 10^{-5}$	1×10^{-4}
RND (WES vs. UTA)	0.382 ± 0.016	0.222 ± 0.009	0.178 ± 0.001	$< 1 \times 10^{-5}$	$< 1 \times 10^{-5}$
F_{ST} (COL vs. NOR)	0.163 ± 0.006	0.173 ± 0.008	0.223 ± 0.001	$< 1 \times 10^{-5}$	$< 1 \times 10^{-5}$
F_{ST} (COL vs. UTA)	0.084 ± 0.004	0.091 ± 0.005	0.138 ± 0.001	$< 1 \times 10^{-5}$	$< 1 \times 10^{-5}$
F_{ST} (UTA vs. NOR)	0.13 ± 0.005	0.14 ± 0.007	0.169 ± 0.001	$< 1 \times 10^{-5}$	0.0035
F_{ST} (WES vs. COL)	0.259 ± 0.009	0.305 ± 0.011	0.445 ± 0.002	$< 1 \times 10^{-5}$	$< 1 \times 10^{-5}$
F_{ST} (WES vs. NOR)	0.274 ± 0.01	0.328 ± 0.013	0.566 ± 0.002	$< 1 \times 10^{-5}$	$< 1 \times 10^{-5}$
F_{ST} (WES vs. UTA)	0.269 ± 0.009	0.329 ± 0.011	0.504 ± 0.002	$< 1 \times 10^{-5}$	$< 1 \times 10^{-5}$
**LG5p**					
π (COL)	4.472 ± 0.288	3.016 ± 0.148	1.743 ± 0.011	$< 1 \times 10^{-5}$	$< 1 \times 10^{-5}$
π (NOR)	3.41 ± 0.249	1.982 ± 0.143	0.965 ± 0.008	$< 1 \times 10^{-5}$	$< 1 \times 10^{-5}$
π (UTA)	5.331 ± 0.333	3.667 ± 0.193	1.707 ± 0.013	$< 1 \times 10^{-5}$	$< 1 \times 10^{-5}$
π (WES)	3.941 ± 0.253	2.531 ± 0.127	1.185 ± 0.009	$< 1 \times 10^{-5}$	$< 1 \times 10^{-5}$
Scaled π (COL)	0.328 ± 0.026	0.21 ± 0.013	0.131 ± 0.001	$< 1 \times 10^{-5}$	$< 1 \times 10^{-5}$
Scaled π (NOR)	0.261 ± 0.025	0.136 ± 0.011	0.073 ± 0.001	$< 1 \times 10^{-5}$	$< 1 \times 10^{-5}$
Scaled π (UTA)	0.374 ± 0.028	0.252 ± 0.016	0.126 ± 0.001	$< 1 \times 10^{-5}$	$< 1 \times 10^{-5}$
Scaled π (WES)	0.285 ± 0.022	0.189 ± 0.015	0.09 ± 0.001	$< 1 \times 10^{-5}$	$< 1 \times 10^{-5}$
Tajima's *D* (COL)	-0.586 ± 0.079	-0.835 ± 0.071	-0.987 ± 0.007	$< 1 \times 10^{-5}$	0.0685
Tajima's *D* (NOR)	0.052 ± 0.107	-0.245 ± 0.144	-0.456 ± 0.013	$< 1 \times 10^{-5}$	0.1652
Tajima's *D* (UTA)	-0.447 ± 0.076	-0.642 ± 0.076	-1.2 ± 0.009	$< 1 \times 10^{-5}$	$< 1 \times 10^{-5}$
Tajima's *D* (WES)	-0.05 ± 0.082	-0.285 ± 0.085	-0.835 ± 0.009	$< 1 \times 10^{-5}$	$< 1 \times 10^{-5}$
Fay & Wu's *H* (COL)	-0.207 ± 0.029	-0.226 ± 0.032	-0.15 ± 0.003	0.0234	0.014
Fay & Wu's *H* (NOR)	-0.539 ± 0.062	-0.622 ± 0.088	-0.635 ± 0.007	0.128	0.8704

Fay & Wu's *H* (UTA)	-0.221 ± 0.032	-0.269 ± 0.039	-0.276 ± 0.004	0.1098	0.8611
Fay & Wu's *H* (WES)	-0.499 ± 0.045	-0.626 ± 0.054	-0.912 ± 0.006	< 1 x 10^{-5}	< 1 x 10^{-5}
Gene density	4195.617 ± 271.134	4178.185 ± 334.597	4560.753 ± 35.436	0.2531	0.3319
d_{XY} (COL vs. NOR)	3.953 ± 0.232	2.467 ± 0.163	1.472 ± 0.009	< 1 x 10^{-5}	< 1 x 10^{-5}
d_{XY} (COL vs. UTA)	4.419 ± 0.271	2.692 ± 0.149	1.622 ± 0.01	< 1 x 10^{-5}	< 1 x 10^{-5}
d_{XY} (UTA vs. NOR)	4.332 ± 0.271	2.568 ± 0.187	1.338 ± 0.009	< 1 x 10^{-5}	< 1 x 10^{-5}
d_{XY} (WES vs. COL)	4.71 ± 0.285	2.914 ± 0.119	2.31 ± 0.013	< 1 x 10^{-5}	7 x 10^{-4}
d_{XY} (WES vs. NOR)	4.531 ± 0.267	2.828 ± 0.151	2.125 ± 0.012	< 1 x 10^{-5}	2 x 10^{-4}
d_{XY} (WES vs. UTA)	5.36 ± 0.316	3.242 ± 0.144	2.537 ± 0.014	< 1 x 10^{-5}	0.0012
RND (COL vs. NOR)	0.285 ± 0.02	0.162 ± 0.014	0.106 ± 0.001	< 1 x 10^{-5}	< 1 x 10^{-5}
RND (COL vs. UTA)	0.299 ± 0.02	0.17 ± 0.011	0.114 ± 0.001	< 1 x 10^{-5}	< 1 x 10^{-5}
RND (UTA vs. NOR)	0.302 ± 0.022	0.166 ± 0.015	0.094 ± 0.001	< 1 x 10^{-5}	< 1 x 10^{-5}
RND (WES vs. COL)	0.328 ± 0.022	0.186 ± 0.01	0.166 ± 0.001	< 1 x 10^{-5}	0.1163
RND (WES vs. NOR)	0.326 ± 0.023	0.183 ± 0.015	0.155 ± 0.001	< 1 x 10^{-5}	0.0282
RND (WES vs. UTA)	0.364 ± 0.024	0.203 ± 0.011	0.178 ± 0.001	< 1 x 10^{-5}	0.0758
F_{ST} (COL vs. NOR)	0.171 ± 0.013	0.201 ± 0.02	0.223 ± 0.001	1 x 10^{-4}	0.2537
F_{ST} (COL vs. UTA)	0.078 ± 0.006	0.087 ± 0.01	0.138 ± 0.001	< 1 x 10^{-5}	2 x 10^{-4}
F_{ST} (UTA vs. NOR)	0.134 ± 0.009	0.147 ± 0.015	0.169 ± 0.001	0.0049	0.2163
F_{ST} (WES vs. COL)	0.243 ± 0.013	0.27 ± 0.018	0.445 ± 0.002	< 1 x 10^{-5}	< 1 x 10^{-5}
F_{ST} (WES vs. NOR)	0.298 ± 0.018	0.347 ± 0.025	0.566 ± 0.002	< 1 x 10^{-5}	< 1 x 10^{-5}
F_{ST} (WES vs. UTA)	0.237 ± 0.015	0.261 ± 0.018	0.504 ± 0.002	< 1 x 10^{-5}	< 1 x 10^{-5}
**LG7p**					
π (COL)	4.54 ± 0.258	3.072 ± 0.117	1.743 ± 0.011	< 1 x 10^{-5}	< 1 x 10^{-5}
π (NOR)	3.064 ± 0.193	1.998 ± 0.092	0.965 ± 0.008	< 1 x 10^{-5}	< 1 x 10^{-5}
π (UTA)	5.423 ± 0.298	3.637 ± 0.135	1.707 ± 0.013	< 1 x 10^{-5}	< 1 x 10^{-5}
π (WES)	4.194 ± 0.202	2.637 ± 0.1	1.185 ± 0.009	< 1 x 10^{-5}	< 1 x 10^{-5}
Scaled π (COL)	0.359 ± 0.029	0.231 ± 0.012	0.131 ± 0.001	< 1 x 10^{-5}	< 1 x 10^{-5}
Scaled π (NOR)	0.243 ± 0.021	0.149 ± 0.008	0.073 ± 0.001	< 1 x 10^{-5}	< 1 x 10^{-5}
Scaled π (UTA)	0.407 ± 0.029	0.266 ± 0.014	0.126 ± 0.001	< 1 x 10^{-5}	< 1 x 10^{-5}
Scaled π (WES)	0.328 ± 0.023	0.188 ± 0.011	0.09 ± 0.001	< 1 x 10^{-5}	< 1 x 10^{-5}
Tajima's *D* (COL)	-0.619 ± 0.066	-0.878 ± 0.059	-0.987 ± 0.007	< 1 x 10^{-5}	0.0831
Tajima's *D* (NOR)	0.135 ± 0.093	-0.117 ± 0.1	-0.456 ± 0.013	< 1 x 10^{-5}	0.0014
Tajima's *D* (UTA)	-0.48 ± 0.066	-0.741 ± 0.062	-1.2 ± 0.009	< 1 x 10^{-5}	< 1 x 10^{-5}
Tajima's *D* (WES)	0.106 ± 0.068	-0.141 ± 0.084	-0.835 ± 0.009	< 1 x 10^{-5}	< 1 x 10^{-5}
Fay & Wu's *H* (COL)	-0.157 ± 0.022	-0.199 ± 0.026	-0.15 ± 0.003	0.7006	0.0292
Fay & Wu's *H* (NOR)	-0.532 ± 0.055	-0.614 ± 0.066	-0.635 ± 0.007	0.0425	0.7098
Fay & Wu's *H* (UTA)	-0.194 ± 0.03	-0.257 ± 0.038	-0.276 ± 0.004	0.0032	0.5596
Fay & Wu's *H* (WES)	-0.375 ± 0.04	-0.471 ± 0.052	-0.912 ± 0.006	< 1 x 10^{-5}	< 1 x 10^{-5}
Gene density	3730.91 ± 189.84	3396.622 ± 219.057	4560.753 ± 35.436	0.0016	< 1 x 10^{-5}
d_{XY} (COL vs. NOR)	3.657 ± 0.171	2.524 ± 0.102	1.472 ± 0.009	< 1 x 10^{-5}	< 1 x 10^{-5}
d_{XY} (COL vs. UTA)	4.24 ± 0.213	2.73 ± 0.103	1.622 ± 0.01	< 1 x 10^{-5}	< 1 x 10^{-5}
d_{XY} (UTA vs. NOR)	4.019 ± 0.204	2.627 ± 0.109	1.338 ± 0.009	< 1 x 10^{-5}	< 1 x 10^{-5}
d_{XY} (WES vs. COL)	4.48 ± 0.207	2.998 ± 0.105	2.31 ± 0.013	< 1 x 10^{-5}	< 1 x 10^{-5}
d_{XY} (WES vs. NOR)	4.286 ± 0.206	2.92 ± 0.114	2.125 ± 0.012	< 1 x 10^{-5}	< 1 x 10^{-5}
d_{XY} (WES vs. UTA)	4.988 ± 0.215	3.338 ± 0.119	2.537 ± 0.014	< 1 x 10^{-5}	< 1 x 10^{-5}
RND (COL vs. NOR)	0.272 ± 0.016	0.174 ± 0.01	0.106 ± 0.001	< 1 x 10^{-5}	< 1 x 10^{-5}
RND (COL vs. UTA)	0.307 ± 0.018	0.184 ± 0.01	0.114 ± 0.001	< 1 x 10^{-5}	< 1 x 10^{-5}
RND (UTA vs. NOR)	0.286 ± 0.017	0.177 ± 0.01	0.094 ± 0.001	< 1 x 10^{-5}	< 1 x 10^{-5}
RND (WES vs. COL)	0.329 ± 0.018	0.202 ± 0.01	0.166 ± 0.001	< 1 x 10^{-5}	8 x 10^{-4}
RND (WES vs. NOR)	0.31 ± 0.017	0.199 ± 0.011	0.155 ± 0.001	< 1 x 10^{-5}	< 1 x 10^{-5}
RND (WES vs. UTA)	0.356 ± 0.019	0.223 ± 0.012	0.178 ± 0.001	< 1 x 10^{-5}	4 x 10^{-4}
F_{ST} (COL vs. NOR)	0.192 ± 0.009	0.215 ± 0.012	0.223 ± 0.001	0.0028	0.5865
F_{ST} (COL vs. UTA)	0.08 ± 0.004	0.084 ± 0.004	0.138 ± 0.001	< 1 x 10^{-5}	< 1 x 10^{-5}
F_{ST} (UTA vs. NOR)	0.15 ± 0.007	0.163 ± 0.01	0.169 ± 0.001	0.0402	0.6099

F_{ST} (WES vs. COL)	0.218 ± 0.012	0.236 ± 0.015	0.445 ± 0.002	$< 1 \times 10^{-5}$	$< 1 \times 10^{-5}$
F_{ST} (WES vs. NOR)	0.304 ± 0.012	0.331 ± 0.014	0.566 ± 0.002	$< 1 \times 10^{-5}$	$< 1 \times 10^{-5}$
F_{ST} (WES vs. UTA)	0.207 ± 0.01	0.234 ± 0.014	0.504 ± 0.002	$< 1 \times 10^{-5}$	$< 1 \times 10^{-5}$

[a] d_{XY} and π are in unit of 10^{-3}, and gene density is total length of coding regions (bp) per 20-kb window.

Table S10 ANOVA (offset from reference group) test of recombiantion rate between BLS regions and background

Source	df	*F*	*P*
WES			
Model	3	1.0331	0.3766
Error	7221		
C. Total	7224		
NOR			
Model	3	2.4242	0.0638
Error	6676		
C. Total	6679		
UTA			
Model	3	5.7599	0.0006
Error	7198		
C. Total	7201		
COL			
Model	3	34.7665	<0.0001
Error	7287		
C. Total	7290		
COL1			
Model	3	2.2534	0.080
Error	7233		
C. Total	7236		
COL2			
Model	3	53.7963	<0.0001
Error	7282		
C. Total	7285		

note: in each model, background was used as reference group.

Table S11 ANOVA test of scaled diversity (π) BLS regions and background by controlling recombination rate (recombination) and gene density (gene_density).

Group	Region	Source	df	*F*	*P*
UTA	LG1	region	1	1180.69	<2e-16
		recombination	1	171.65	<2e-16
		gene_density	1	69.24	<2e-16
		Residuals	6909		
	LG5	region	1	377.11	<2e-16
		recombination	1	81.94	<2e-16
		gene_density	1	121.45	<2e-16
		Residuals	6733		
	LG7	region	1	715.1	<2e-16
		recombination	1	79.46	<2e-16
		gene_density	1	94.68	<2e-16
		Residuals	6783		
WES	LG1	region	1	1525.9	<2e-16
		recombination	1	682	<2e-16
		gene_density	1	7.25	0.00711
		Residuals	6931		
	LG5	region	1	427.6	<2e-16
		recombination	1	574.3	<2e-16
		gene_density	1	20.7	5.45E-06
		Residuals	6755		
	LG7	region	1	928.16	<2e-16
		recombination	1	420.06	<2e-16
		gene_density	1	12.25	0.000469
		Residuals	6804		
COL	LG1	region	1	1145.7	<2e-16
		recombination	1	330.48	<2e-16
		gene_density	1	45.17	1.94E-11
		Residuals	6998		
	LG5	region	1	307.12	<2e-16
		recombination	1	227.64	<2e-16
		gene_density	1	86.59	<2e-16
		Residuals	6821		
	LG7	region	1	593.89	<2e-16
		recombination	1	296.03	<2e-16
		gene_density	1	64.43	1.17E-15
		Residuals	6871		
NOR	LG1	region	1	1267.127	<2e-16
		recombination	1	117.549	<2e-16
		gene_density	1	5.036	0.0249
		Residuals	6393		
	LG5	region	1	404.72	<2e-16
		recombination	1	58.59	2.24E-14
		gene_density	1	28.88	7.98E-08
		Residuals	6217		
	LG7	region	1	485.01	<2e-16
		recombination	1	69.88	<2e-16
		gene_density	1	20.26	6.88E-06
		Residuals	6264		

Table S12 Number and size of F_{ST}-islands in each *B. stricta* group pairs.

Group Pairs	No. of 20-kb outliers	No. of combined outliers*	Mean size (kb)*	Max size (kb)*
WES vs. COL	163	125	26.1	100
WES vs. NOR	22	19	23.2	40
WES vs. UTA	85	72	23.6	60
COL vs. NOR	306	225	27.2	120
COL vs. UTA	349	249	28	140
UTA vs. NOR	352	239	29.5	140

*adjacent outliers were jonted into single combined outliers

Table S13 ANOVA test of F_{ST} between F_{ST}-islands and genomic background by controlling for recombiantion rate (rec1 and rec2) and gene density (gene_density).

Source	df	*F*	*P*
WES vs. COL			
region	1	977.25	2.00E-16
rec1	1	99.95	2.00E-16
rec2	1	83.05	2.00E-16
gene_density	1	59.1	1.71E-14
Residuals	6786		
WES vs. NOR			
region	1	86.426	2.00E-16
rec1	1	133.261	2.00E-16
rec2	1	4.157	0.0415
gene_density	1	65.28	7.76E-16
Residuals	6126		
WES vs. UTA			
region	1	402.592	2.00E-16
rec1	1	127.545	2.00E-16
rec2	1	0.436	0.509
gene_density	1	64.943	9.06E-16
Residuals	6710		
COL vs.NOR			
region	1	3624.895	2.00E-16
rec1	1	0.326	0.56833
rec2	1	8.215	0.00417
gene_density	1	25.484	4.59E-07
Residuals	6194		
COL vs. UTA			
region	1	5123.269	2.00E-16
rec1	1	10.267	0.00136
rec2	1	0.112	0.737524
gene_density	1	14.02	0.000182
Residuals	6820		
UTA vs. NOR			
region	1	4511.748	2.00E-16
rec1	1	27.274	1.82E-07
rec2	1	0.183	0.669
gene_density	1	0.368	0.544
Residuals	6136		

Table S14 GO categories overrepresented for genes located in F_{ST}-islands

GO term	Ontology	Description	*P*-value	FDR
**COL vs. NOR**				
GO:0005986	P	sucrose biosynthetic process	4.30E-05	0.028
GO:0046351	P	disaccharide biosynthetic process	9.40E-05	0.031
GO:0009312	P	oligosaccharide biosynthetic process	0.00022	0.047
GO:0050897	F	cobalt ion binding	4.10E-05	0.0088
GO:0016157	F	sucrose synthase activity	0.00021	0.023
GO:0015114	F	phosphate ion transmembrane transporter activity	0.00058	0.041
**COL vs. WES**				
GO:0008187	F	poly-pyrimidine tract binding	0.00016	0.012
GO:0008266	F	poly(U) RNA binding	0.00011	0.012
**WES vs. UTA**				
GO:0071705	P	nitrogen compound transport	1.60E-05	0.0048

NOTE, No GO categories were over-represented for genes located in islands of the other three comparisons, COL vs. UTA, WES vs. NOR and UTA vs. NOR.

Table S15 Error rate as a function of sequencing depth based on reads from accessions LTM and SAD12.

SampleID	Depth	TP	FHET	FHOM	MISS	TPR (%)	MR (%)	FR (%)
LTM	1	909674	60	825	3214837	99.9028	77.9280	0.0972
LTM	2	1617664	246	850	2506635	99.9323	60.7611	0.0677
LTM	2.5	2030457	582	694	2093662	99.9372	50.7506	0.0628
LTM	5	3564980	7925	157	552332	99.7738	13.3886	0.2262
LTM	10	3984018	16271	39	125067	99.5923	3.0316	0.4077
LTM	15	4044281	18279	30	62805	99.5493	1.5224	0.4507
LTM	40	4080454	20007	18	24916	99.5116	0.6040	0.4884
SAD12	1	1036588	1	13150	3075657	98.7472	74.5542	1.2528
SAD12	2	1818442	8	19683	2287262	98.9287	55.4435	1.0713
SAD12	2.5	2256940	38	22182	1846235	99.0251	44.7529	0.9749
SAD12	5	3736172	744	22444	366035	99.3832	8.8727	0.6168
SAD12	10	4035425	2766	14805	72399	99.5665	1.7550	0.4335
SAD12	15	4070682	3293	10466	40955	99.6631	0.9928	0.3369
SAD12	40	4098076	2807	4222	20290	99.8288	0.4918	0.1712

TP, True positive; FHET, false heterozygote; FHOM, false homozygote; MISS, missing loci.

TPR, true positive rate, defined as TP/(TP + FHET + FHOM),

FR, false discovery rate, defined as (FHET + FHOM)/(TP + FHET + FHOM)

MR, missing rate, defined as MISS/(TP + FHET + FHOM + MISS)
